# Damage-responsive elements in *Drosophila* regeneration

**DOI:** 10.1101/gr.233098.117

**Published:** 2018-12

**Authors:** Elena Vizcaya-Molina, Cecilia C. Klein, Florenci Serras, Rakesh K. Mishra, Roderic Guigó, Montserrat Corominas

**Affiliations:** 1 Departament de Genètica, Microbiologia i Estadística, Facultat de Biologia and Institut de Biomedicina (IBUB), Universitat de Barcelona, Barcelona 08028, Catalonia, Spain;; 2 Centre for Genomic Regulation (CRG), The Barcelona Institute of Science and Technology, Barcelona 08003, Catalonia, Spain;; 3 The Centre for Cellular and Molecular Biology (CCMB), Hyderabad 500007, India;; 4 Universitat Pompeu Fabra (UPF), Barcelona 08003, Catalonia, Spain

## Abstract

One of the most important questions in regenerative biology is to unveil how and when genes change expression and trigger regeneration programs. The resetting of gene expression patterns during response to injury is governed by coordinated actions of genomic regions that control the activity of multiple sequence-specific DNA binding proteins. Using genome-wide approaches to interrogate chromatin function, we here identify the elements that regulate tissue recovery in *Drosophila* imaginal discs, which show a high regenerative capacity after genetically induced cell death. Our findings indicate there is global coregulation of gene expression as well as a regeneration program driven by different types of regulatory elements. Novel enhancers acting exclusively within damaged tissue cooperate with enhancers co-opted from other tissues and other developmental stages, as well as with endogenous enhancers that show increased activity after injury. Together, these enhancers host binding sites for regulatory proteins that include a core set of conserved transcription factors that control regeneration across metazoans.

Research in regenerative medicine seeks to identify the genes and regulatory regions that control tissue regeneration. The capacity to regenerate varies greatly, not only between species, but also between tissues and organs, as well as from one developmental stage to another in the same species. Organisms such as planarians, cnidarians, and sponges have a high capacity to regenerate, whereas more complex animals, such as mammals, have a more restricted potential to regenerate (Nacu and Tanaka 2011; Tanaka and Reddien 2011; Chen and Poss 2017; Slack 2017).

During tissue and organ regeneration, certain cells detect damage and switch their transcriptional programs to reconstruct the tissue; this involves spatial and temporal regulation of gene expression (Maurange et al. 2006; Katsuyama et al. 2015; Kang et al. 2016; Rodius et al. 2016). In recent years, many studies of animal models have focused on analyzing how the regeneration process begins and the early signals that initiate it. As a result, several genes and signaling pathways have been demonstrated to be required during the process (Chen and Poss 2017; Hariharan and Serras 2017). However, a complete understanding of regeneration requires discerning how the signals are integrated into the genome to induce changes in transcription and chromatin dynamics throughout the entire process. Recent studies in zebrafish have characterized cell-type–specific regulatory elements for heart regeneration (Kang et al. 2016; Goldman et al. 2017). Nevertheless, an integrated view of other regenerative systems at the genome-wide level is necessary to shed light on the combinatorial regulatory network that is required to induce regeneration.

*Drosophila* imaginal discs, the epithelial larval precursors of adult structures, present a high capacity to regenerate after damage (Worley et al. 2012; Jaszczak and Halme 2016; Hariharan and Serras 2017). In this study, we characterize the gene expression profiles and the map of regulatory elements that respond to cell death-induced regeneration throughout the recovery process of *Drosophila* wing imaginal discs.

## Results

### Regeneration: a burst of active transcription

**Figure 1.**
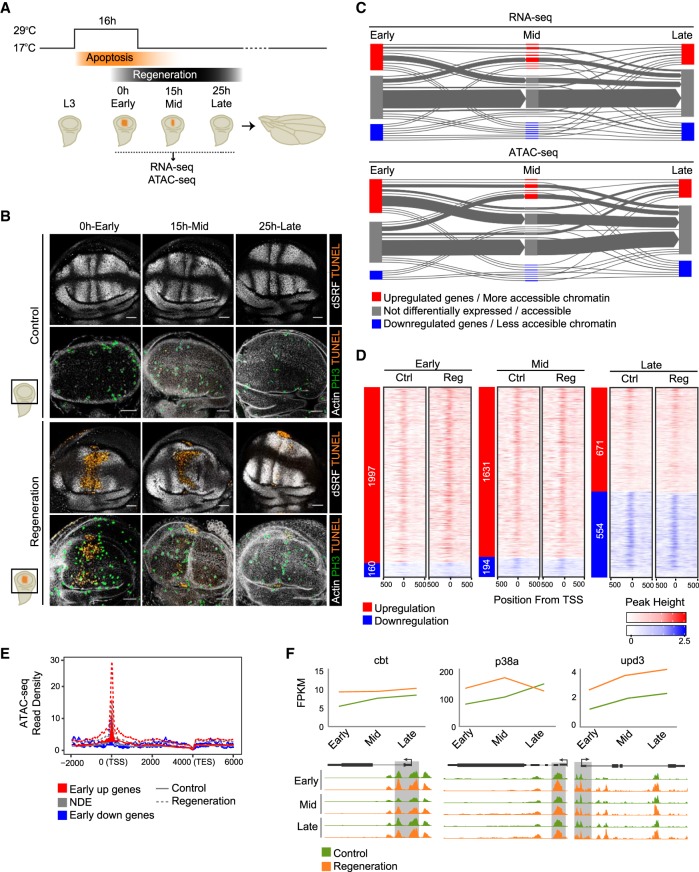
Differentially expressed genes and accessible chromatin after induction of cell death. (*A*) Experimental design. Flies were raised at 17°C until the eighth day (192 h) after egg laying (equivalent to third instar larva or L3). Then they were moved to 29°C for 16 h to induce apoptosis triggered by *rpr* (*reaper*), specifically in the *spalt major* (*salm*) domain of the wing pouch (orange region), and then back to 17°C to switch off *rpr* and allow the tissue to regenerate. RNA-seq and ATAC-seq samples were collected immediately after switching off *rpr* (0 h: early), or at successive time points (15 h: mid; and 25 h: late). Controls without having undergone *rpr* expression were treated in parallel. (*B*) Confocal images of wing discs stained with *Drosophila* serum response factor (bs, also known as DSRF) antibody and actin to visualize the patterning, TUNEL assay to detect cell death, and H3P antibody to detect mitosis. (*C*) Flux plot showing RNA-seq dynamics (DE genes) and ATAC-seq dynamics (differential accessible regions) at the different time points. Each line represents a set of genes or accessible regions that behave in the same way over time. The line width denotes the number of genes or accessible regions. (*D*) Heatmaps showing ATAC-seq signal around ±500 bp from the transcription start site (TSS) of protein-coding DE genes at each time point. Sites are ordered by up-regulation and down-regulation (shown on the *left*) and by gene expression, based on regeneration samples. (*E*) Aggregation plot showing ATAC-seq read density at the early stage (control and regeneration) for each set of DE genes (up-regulated, nondifferentially expressed [NDE], and down-regulated). The TSSs of up-regulated genes show the highest number of ATAC-seq reads in regeneration. (*F*) Expression profile (FPKM) of *cbt, p83a*, and *upd1* over time, in control and regeneration (*top*). Genome Browser screenshots depicting ATAC-seq peaks at the core promoter of *cbt*, *p83a*, and *upd1*, in control and regeneration over time (*bottom*).

To elucidate the transcriptional regulatory network that controls tissue regeneration, we characterized the gene expression profiles (by RNA-seq) and the map of accessible regions (by ATAC-seq) associated with the response to damage in *Drosophila melanogaster* wing imaginal discs (for a workflow, see Supplemental Figs. S1, S2). Genetic ablation was induced by expression of the proapoptotic gene *reaper* (*rpr*), controlled in time and space by a temperature shift. Briefly, flies were raised at 17°C until the eighth day (192 h) after egg laying (equivalent to third larval instar or L3). They were then moved to 29°C for 16 h to induce apoptosis triggered by *rpr*, specifically in the *spalt major* (*salm*) domain of the wing pouch, and then back to 17°C to switch off *rpr* and enable tissue regeneration (Bergantiños et al. 2010a,b; Repiso et al. 2013; Santabárbara-Ruiz et al. 2015). We collected samples at three time points after the induction of apoptosis (Fig. 1A). The first time point corresponds to immediately after switching off *rpr* (0 h: early), when some of the early signals are known to act (Smith-Bolton et al. 2009; Bergantiños et al. 2010a; Repiso et al. 2013; Santabárbara-Ruiz et al. 2015). At this stage, apoptotic cells were extruded from the epithelium, patterning was disrupted, and mitotic cells localized mostly at the edges of the wound (Fig. 1B). The second time point corresponds to an intermediate step or 15 h after apoptosis (15 h: mid). At this stage, patterning had not yet recovered, although living cells almost closed the wound. Moreover, a localized mitotic zone was also found at the edges of the wound closure (Fig. 1B). Finally, the third selected time point corresponds to 25 h after apoptosis (25 h: late). The wound was completely closed, and both size and patterning were mostly reconstructed (Fig. 1B). Discs lacking *rpr* expression, but kept at the same temperatures, were used as controls. We performed pairwise comparisons between control and regenerating discs at each time point (Supplemental Table S1).

RNA-seq analysis showed the highest number of differentially expressed (DE) protein-coding genes at the early stage (1997), most of which (92%) were up-regulated in the regeneration samples (Supplemental Fig. S3). This number decreased over time, correlating with the recovery of tissue morphology (Fig. 1B,C; Supplemental Fig. S3). A total of 28% of the genes that were up-regulated at the early stage were also up-regulated at other stages. In keeping with this, the analysis of ATAC-seq data showed that the number of regions that were more accessible in injured than in control samples was highest during early regeneration and decreased over time, correlating with the activation of transcription during the initial steps (Fig. 1C; Supplemental Fig. S4). We next examined the core promoter region (CP; ±100 bp from the transcription start site [TSS]) of DE genes at the early stage and found it was accessible in all the expressed genes (Fig. 1D). As expected, we found increased accessibility around the TSS of up-regulated genes at this early stage (Fig. 1E).

Among the up-regulated genes, we found *unpaired 3* (*upd3*), *Jun-related antigen* (*Jra*), *cabut* (*cbt*), and *p38a MAP kinase* (*p38a*) (Fig. 1F), which are known to be required only in a few cells around the wound after cell death and physical injury (Blanco et al. 2010; Katsuyama et al. 2015; Santabárbara-Ruiz et al. 2015). We also found up-regulation of genes, such as *Atf3*, *mol*, *fru*, *LamC*, and *pigs*, which had been identified and validated in a previous transcriptome study of wing disc regeneration (Khan et al. 2017). This demonstrated that our approach was sensitive enough to identify changes affecting small numbers of cells within the population studied.

### Global coregulation of the expression of signaling pathway genes throughout regeneration

**Figure 2.**
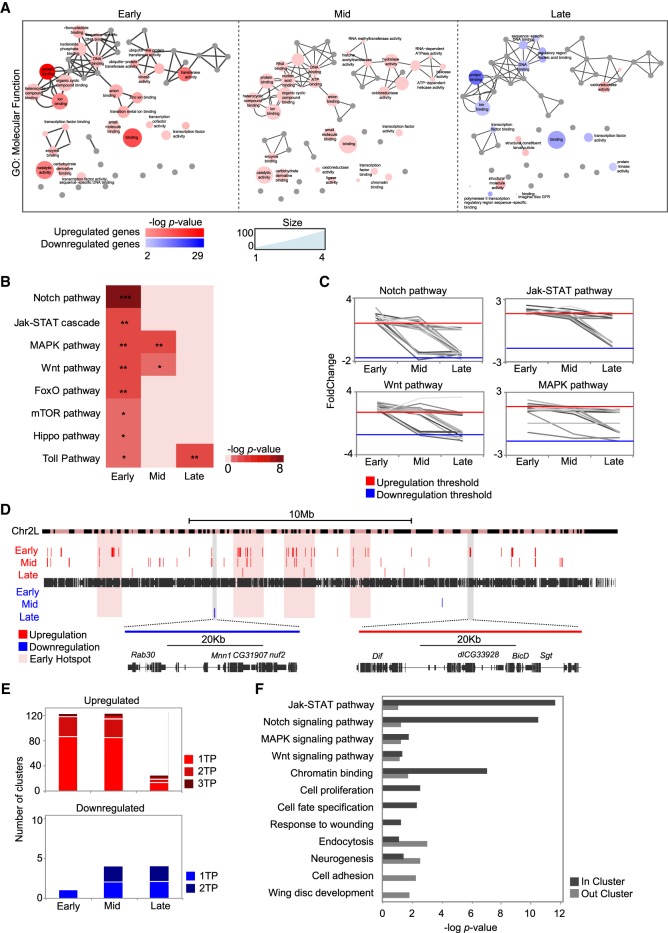
Genomic clustering of differentially expressed genes. (*A*) Gene Ontology term enrichment of differentially expressed genes at successive time points visualized by ReviGO. The size of the circles denotes the number of genes; circle color indicates the *P*-value of each term. Highly similar GO terms are linked by edges in the graph. (*B*) Heatmap of pathway enrichment in the set of up-regulated genes at each time point. The level of significance is denoted: (*) *P* < 0.05; (**) *P* < 10^−2^; (***) *P* < 10^−3^. (*C*) Line plots showing expression changes over time of genes that belong to signaling pathways significantly enriched in regeneration. Expression is shown as fold change between control and regeneration at each time point. Each gene is plotted as a single line. (*D*) Genomic map of clusters of differentially expressed genes on Chromosome 2L. Each red or blue box represents a single cluster; the size of each box denotes the length of the cluster. The magnified regions show one down-regulated cluster (blue, *left*) and one up-regulated cluster (red, *right*). Hotspots at the early stage are highlighted in pink. (*E*) Bar plot showing the number of clusters identified at only one time point, at two time points, and at all three time points. (*F*) Gene Ontology term enrichment for the set of up-regulated genes located inside or outside the clusters at early regeneration. All the categories plotted are significant in at least one group of genes (the absence of a bar denotes no enrichment in that group).

Analysis of DE genes revealed functional enrichment in Gene Ontology (GO) terms related to transcription factor (TF) activity, kinase activity, or DNA binding only at the early stage (Fig. 2A). Moreover, we found a set of 195 TFs induced in regeneration, 68% of which were induced at this early stage (Supplemental Fig. S5).

We found that the pathways already known to play a role in regenerative growth (Hariharan and Serras 2017) were enriched in our set of up-regulated genes after damage; Notch, Jak-STAT, MAPK, and Wnt were the most enriched (Fig. 2B; Supplemental Fig. S6). As with the main burst of active transcription, this enrichment occurred mainly at the early stage. When we analyzed expression of DE genes in each pathway over time, we found similar transcription patterns for several members of the pathway (Fig. 2C; Supplemental Fig. S6).

A number of transcriptome studies have demonstrated that genes with similar patterns of expression are frequently located close to one another in linear genomes (Boutanaev et al. 2002; Sproul et al. 2005; Michalak 2008; Corrales et al. 2017). Hence, we examined the chromosomal distribution of DE genes at all the time points. We identified several clusters of up-regulated genes, mostly at early and mid regeneration (126 and 124, respectively) (Fig. 2D,E; Supplemental Fig. S7; Supplemental Table S2), indicating that large regions, rather than individual genes, may be controlled by the same regulatory elements. Protein-coding genes inside up-regulated clusters showed expression profiles that were more similar over time than those of genes in down-regulated clusters and genes overall (Supplemental Fig. S7). We also observed that some clusters were close to one another, creating genomic hotspots (Fig. 2D; Supplemental Table S2). GO analysis of the clusters at the early time point revealed that genes inside them were significantly linked to signaling pathways, proliferation, and response to wounding, whereas up-regulated genes outside the clusters tended to be more associated with development, cell adhesion, or neurogenesis (Fig. 2F; Supplemental Fig. S8 for the other time points).

### Damage-responsive regulatory elements (DRREs)

**Figure 3.**
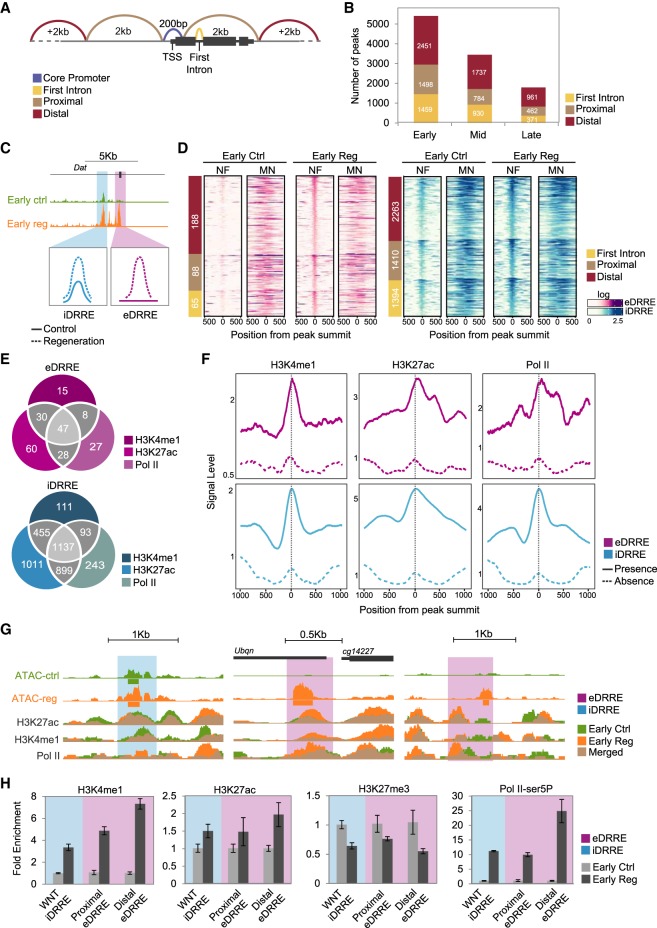
Accessible chromatin landscape after induction of cell death. (*A*) Schematic overview of peak distribution in the genome. (*B*) Bar plot showing the number of more accessible peaks at each time point falling in each genomic region: first intron, proximal, and distal. (*C*) Genome Browser screenshot and schematic drawing of iDRRE and eDRRE. (*D*) Heatmaps showing nucleosome-free (NF) and mononucleosome (MN) enrichment around ±500 bp from the peak summit of DRREs at the early stage of control and regeneration. Sites are ordered by genomic distribution (shown on the *left*), and by peak height based on ATAC-seq regeneration sample. (*E*) Venn diagrams showing the intersection of H3K4me1, H3K27ac, and Pol II at DRREs in regeneration. (*F*) Average profile of H3K4me1, H3K27ac, and Pol II at DRREs. A solid line denotes DRREs with the presence of at least one ChIP-seq signal; and a dashed line denotes the absence of any ChIP-seq signal. (*G*) Genome Browser screenshot showing ATAC-seq and ChIP-seq profiles (control and regeneration) of the DRREs tested by ChIP-qPCR. (*H*) ChIP-qPCR analysis of H3K4me1, H3K27ac, H3K27me3, and Pol II-ser5P on individual DRREs at the early stage. ChIP results are presented as fold change enrichment between control and regeneration. Error bars represent the standard error of the mean from two biological replicates.

We next studied changes in chromatin dynamics that could trigger the regenerative transcriptional profile by analyzing chromatin accessibility. As differences in gene expression mainly correspond to a burst of transcription after damage, we focused on regions that presented higher accessibility in regeneration, compared to controls, and named these regions damage-responsive regulatory elements (DRREs). To further characterize DRREs, we obtained ATAC-seq data for untreated L3 wing imaginal discs, because these represent the basal developmental stage of the tissue we studied (Supplemental Fig. S9). To differentiate accessible regions in the CP from those representing putative enhancers, we classified ATAC-seq peaks according to their position relative to the TSS of the closest gene. Thus we classified regions that become more accessible under damage conditions as being in the CP (±100 bp of the TSS), in the first intron (FI), proximal (±2 kb from the TSS), and distal (more than ± 2 kb away from the TSS) (Fig. 3A; Supplemental Table S3 for peak classification). Despite the number of accessible chromatin regions decreasing over time, we did not observe differences in their genomic distribution, suggesting that the proportions of each type of enhancer are maintained over time (Fig. 3B).

This analysis enabled us to distinguish two types of DRREs, which, following the studies of zebrafish heart regeneration (Goldman et al. 2017), we named *emerging* (open regions detected only after damage [eDRREs] and corresponding to 6.3% of all DRREs at the early stage) and *increasing* (regions already open in early-stage control discs and L3, but displaying increased accessibility after damage [iDRREs], and corresponding to 93.7% of all DRREs at the early stage) (Fig. 3C,D; Supplemental Fig. S10 for the other time points). Among the iDRREs, we found the damage-activated Wnt (WNT) enhancer that has already proven to be crucial in imaginal disc regeneration (Harris et al. 2016), thus once again validating the sensitivity of our method (Supplemental Fig. S10).

Compared to the iDRREs, eDRREs tend to occur more often in distal locations at the early time point (Fig. 3D). We also compared the nucleosome-free (NF) and mononucleosome (MN) fractions from the ATAC-seq experiments, since regions with or without nucleosomes may present different features (Jung et al. 2017). In iDRREs, we detected ATAC-seq reads in the NF region, which was flanked by well-positioned nucleosomes both in control and regeneration samples, whereas for eDRREs, we observed reads in the NF regions only after damage (Fig. 3D; Supplemental Fig. S10 for the other time points).

Certain post-translational modifications of histone residues are predictive of active enhancers, whereas inactive ones are associated with H3K27me3. The modifications that are most commonly found at enhancers are H3K4me1 and H3K27ac (Supek et al. 2011; Calo and Wysocka 2013; Shlyueva et al. 2014; Koenecke et al. 2016; Long et al. 2016). Although highly correlated with enhancer activity, it is currently not clear whether these marks are required for activity or if they are the consequence of the enhancer's activity (Dorighi et al. 2017; Pollex and Furlong 2017; Catarino and Stark 2018). RNA polymerase II (Pol II) occupancy and transcription are also predictive of active enhancers (Catarino and Stark 2018; Mikhaylichenko et al. 2018). To further characterize enhancer features of DRREs, we first took advantage of the available ChIP-seq data for L3 discs on histone modifications (Pérez-Lluch et al. 2015; Loubière et al. 2016) and found that eDRREs, but not iDRREs, showed a positioned nucleosome (histone 3) modified with the repressive mark H3K27me3 (Supplemental Fig. S12). We next performed ChIP-seq analysis on H3K27ac, H3K4me1, and Pol II binding at the early stage and found that 80% of iDRREs and 63% of eDRREs displayed features of active enhancers in regeneration (Fig. 3E–G; Supplemental Figs. S11, S12). Around 30% of eDRREs were only marked by H3K4me1 or H3K27ac, or showed Pol II binding, whereas 14% contained all of them. In the case of iDRREs, 8.1% presented only one feature, in contrast to 20% containing all three. We further confirmed these results by ChIP-qPCR analysis of individual DRREs. We selected the following genomic regions: the WNT enhancer (Harris et al. 2016) as an iDRRE; a proximal eDRRE located inside a cluster of up-regulated genes and 1.5 kb away from the TSS of *CG1422*7; and a distal eDRRE located more than 48 kb away from the nearest up-regulated protein-coding gene: *leucine-rich-repeats and calponin homology domain protein* (*lrch*). We observed a decrease of H3K27me3 as well as an increase of H3K4me1, H3K27ac, and the active form of Pol II phosphorylated in Serine 5 (Pol II-ser5P) in the WNT enhancer (iDRRE) and in both the proximal and distal eDRREs (Fig. 3H). All this analysis indicates that eDRREs are indeed in closed chromatin in L3 wing discs, becoming accessible and acting as enhancers only after damage.

**Figure 4.**
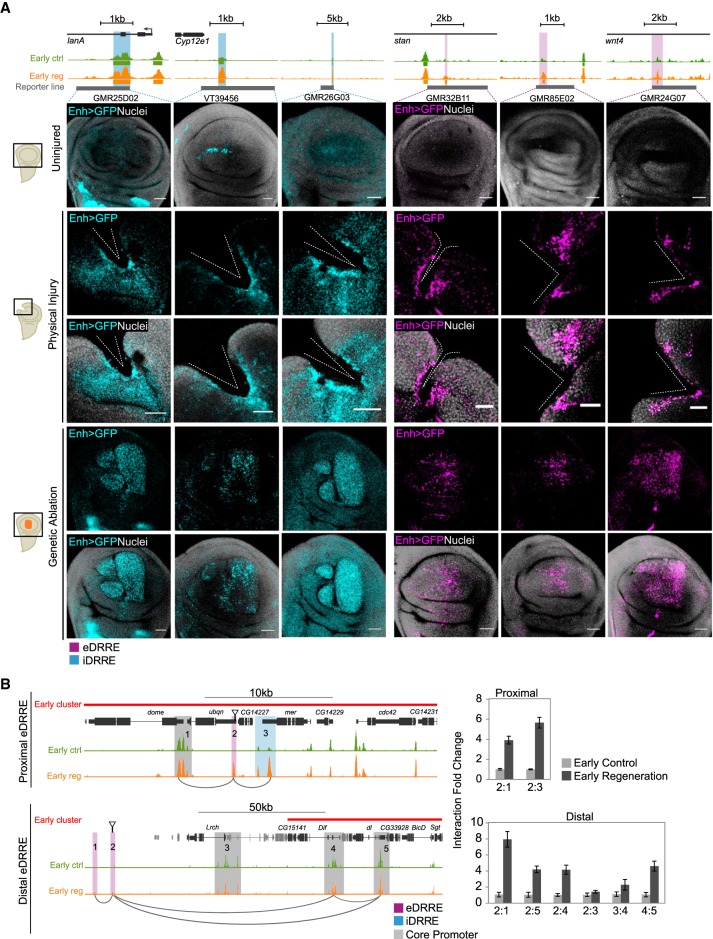
Validation of the activity of DRREs after damage. (*A*) Validation of enhancer regions after induction of cell death or physical damage using reporter lines. Genome Browser screenshot showing the ATAC-seq profile (control and regeneration) at the early stage of regeneration of validated enhancers (highlighted in blue if iDRRE; purple if eDRRE), and the region covered by the reporter line in gray (*top*). Confocal images of wing discs showing enhancer activity as GFP intensity (blue if iDRRE; purple if eDRRE) and nuclei in gray (*bottom*). The injury domain is shown in a schematic drawing on the *right*. In cell death-induced discs, GFP labeling is located in the apical section, where living cells cover the apoptotic zone. (*B*) Genome Browser screenshots at the early stage highlighting regions used for 3C analysis (*left*). Arrows indicate the eDRRE peaks used as bait. qPCR-3C analysis showing interaction levels between eDRREs and both CP and iDRRE (*right*). 3C results are presented as the fold change of the interaction between control and regeneration. Error bars represent the standard error of the mean from two biological replicates. Positive interactions after damage are marked as connectors in the Genome Browser screenshot.

A property of enhancers is their capacity to retain transcription-activating functions outside their endogenous contexts. To further confirm the damage-induced enhancer activity of DRREs in vivo, we used reporter lines and tested them with different types of injury. Fly lines containing DRREs cloned upstream of a Gal4 protein and *UAS-GFP* constructs were subjected to physical injury or genetic ablation using the double transcriptional transactivator, which combines the *UAS-Gal4* system to drive the enhancer activity and the *sal^E/Pv^-LHG lexO-rp*r to induce apoptosis (Santabárbara-Ruiz et al. 2015). Under both conditions, we detected increased or ectopic GFP expression in the damaged zone for iDRREs. Meanwhile, for eDRREs, we only detected GFP expression in the injured region (Fig. 4A; Supplemental Fig. S13). These results confirmed the occurrence of bona fide enhancers responding to injury.

Spatial chromatin organization connects active enhancers to target promoters in *cis* to regulate gene expression (Dekker et al. 2013; Rowley and Corces 2016; Cubeñas-Potts et al. 2017; Schwartz and Cavalli 2017). Thus, we studied whether eDRREs could contact accessible regions (CP and iDRREs) located in up-regulated genes. We selected the same proximal and distal eDRREs tested for enhancer features by ChIP-qPCR, and performed 3C-qPCR analysis at the early stage. Regions already proven to establish contact in L3 wing discs (Bieli et al. 2015) were used as control (Supplemental Fig. S13). We detected interactions between a proximal eDRRE, and both the CP of *domeless* (*dome*) and a proximal iDRRE located at the transcription-ending site of *merlin* (*mer*), within the same cluster of coregulated genes (Fig. 4B). We also detected physical contact between a distal eDRRE and the CPs of the *dorsal-related immunity factor* (*Dif*) and *CG33928* genes, which are located within a cluster of up-regulated genes. In contrast, we did not detect any interaction between the same distal eDRRE and the CP of the *lrch* gene, which, in spite of also being up-regulated and closer in the genome to the eDRRE, is outside the cluster. This suggests that the distal eDRRE could specifically regulate the entire cluster (Fig. 4B).

### A specific regeneration regulome

**Figure 5.**
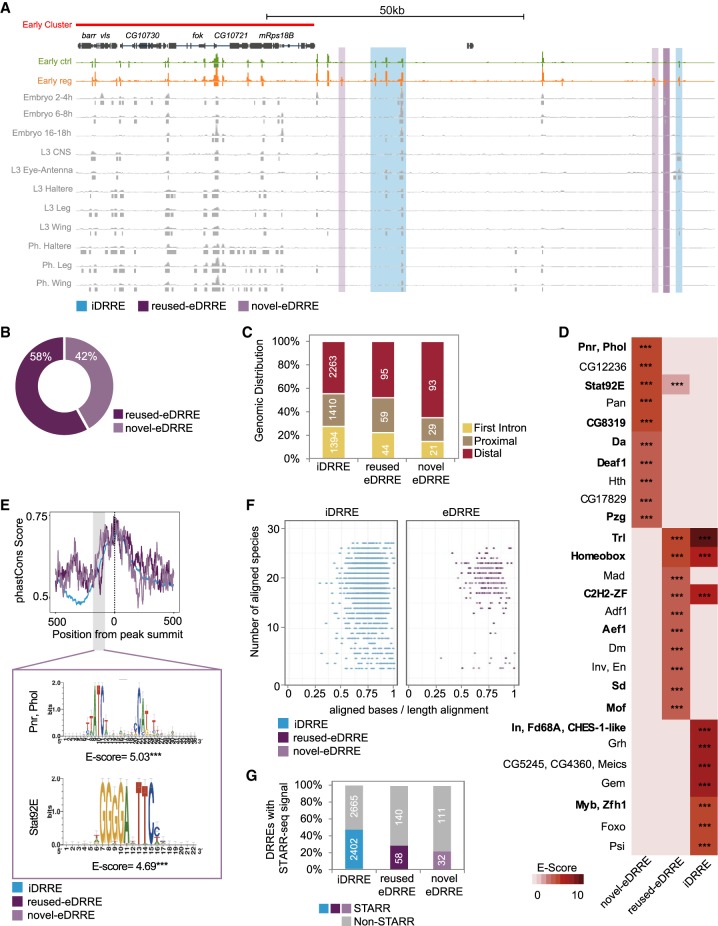
Damage-responding regulatory elements used in other tissues and at other developmental stages. (*A*) Genome Browser screenshot depicting the alignment of iDRREs, reused eDRREs, and novel eDRREs with previously accessible regions identified by FAIRE at different developmental stages and in different tissues. (*B*) Classification of eDRRE usage. FAIRE data for embryo (2–4 h; 6–8 h; 16–18 h), L3 (central nervous system, eye antenna, haltere, leg), and pharate (haltere, leg, wing) are used. eDRREs falling in at least one of the data sets are considered as reused eDRREs. (*C*) Genomic distribution of DRREs: first intron, proximal, and distal. (*D*) Heatmap showing top 10 enriched TF motifs in each DRRE type. Up-regulated TFs are marked in bold. The level of significance is denoted: (***) *P* < 10^−3^. (*E*) Average distribution of PhastCons scores derived from 27 insect species in the DRRE sequences (defined as 500 bp upstream of and downstream from the NF peak summit). The highlighted section represents a more conserved region at −100 bp from the peak summit in novel eDRREs with its motif enrichment. The level of significance is denoted: (***) *P* < 10^−3^. (*F*) Conservation of DRREs across 27 insect species. Each dot corresponds to one independent enhancer. The *y*-axis denotes the number of species that present the conserved enhancer. The *x*-axis represents the percentage of aligned bases per sequence length. (*G*) Percentage of conserved DRREs that are active, according to the STARR-seq technique.

Since enhancers can be used in a context-dependent manner (Nègre et al. 2011; McKay and Lieb 2013; Wei et al. 2016; Erceg et al. 2017), we assessed whether DRREs are involved in other developmental events, regardless of their specific role in wing disc regeneration. We took advantage of chromatin accessibility data from different tissues and stages of fly development (McKay and Lieb 2013). We found that 58% (198) of the eDRREs were already being used in other tissues or across different developmental stages (we renamed these reused eDRREs). The remaining 42% (143) therefore represented novel eDRREs (Fig. 5A,B; Supplemental Fig. S14), a class of enhancers that is probably regeneration specific. We observed that more novel eDRREs tend to be distal within the genome than iDRREs and reused eDRREs (65% compared to 45% and 48%, respectively, at the early stage) (Fig. 5C; Supplemental Table S4 for reusage analysis; Supplemental Fig. S15 for the other time points). To further confirm the usage of reused eDRREs in other tissues, we looked for the endogenous activity of reporter lines. We found that in accordance with the comparative analysis, reused eDRREs are also active in some other tissues (Supplemental Fig. S14).

Next, we searched for motif enrichment in DRREs by using an iCIS target (Herrmann et al. 2012) and selected the motifs for which the corresponding TF was expressed in the wing disc (Fig. 5D; Supplemental Table S5 for the other time points). Of these, we found that 52% of TFs putatively binding to eDRREs (whether novel or reused) and 43% of those putatively binding to iDRREs were up-regulated. Moreover, we observed that motifs found in novel eDRREs are not enriched in the other DRREs (Supplemental Table S5). Altogether, novel eDRREs appear to form part of a regulatory program triggered by TFs, which is different from that controlling reused eDRREs and iDRREs.

Finally, because enhancer activity is often deeply conserved (Stark et al. 2007; He et al. 2011; Arnold et al. 2014), we explored whether DRREs are present in other insect species. We first calculated the average phastCons score for each DRRE, excluding the CP, and found a similar pattern of conservation among DRRE types, with the exception that iDRREs are less conserved in the nucleosome region upstream of the peak summit of the NF region. Novel eDRREs present greater conservation around 100 bp upstream of the peak summit (Fig. 5E; Supplemental Fig. S15 for the other time points). When we applied motif discovery to these regions, we found enrichment for Signal-transducer and activator of transcription protein at 92E (Stat92E), pannier (Pnr), and pleiohomeotic like (Phol), which correspond to the most enriched motifs for novel eDRREs (Fig. 5D,E). Next, we calculated the number of species containing DRREs and found that most eDRREs are present in a large number of species, whereas iDRREs tend to be more species specific (Fig. 5F). This suggests that eDRREs might be involved in the core regulation of regeneration pathways common to all insects. Previous work already demonstrated the activity of several of these conserved enhancers in different *Drosophila* species using the STARR-seq technique (Arnold et al. 2014). Taking advantage of those data, we found that ∼40% of iDRREs are active in other *Drosophila* species, whereas enhancer activity has been proven for only 20% of novel eDRREs (Fig. 5G; Supplemental Fig. S16), which is consistent with these enhancers being activated only after damage.

### Conservation of the regeneration regulatory logic

Because regeneration is widespread in nature (Tanaka and Reddien 2011; Vriz et al. 2014; Chen and Poss 2017), we studied the conservation of genes and regulatory regions that trigger regeneration to determine whether there is a core molecular toolkit underlying organ regeneration in metazoans. We performed a comparative study with zebrafish heart and mammalian liver—two organs with the capacity to regenerate in adult organisms and for which transcriptome data similar to those produced here are available.

**Figure 6.**
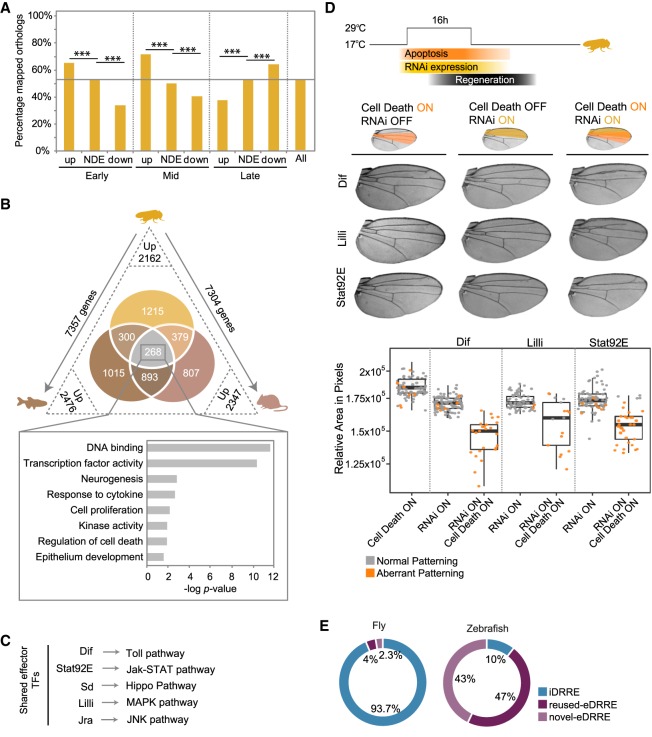
Conservation of regenerative features. (*A*) Bar plot showing the percentage of fly genes with an ortholog in zebrafish, mouse, or both at each time point and for each set of genes (up-regulated, nondifferentially expressed [NDE], and down-regulated). This percentage for all fly protein-coding genes is also shown and highlighted as a horizontal line. (*B*) Venn diagram showing the intersection of up-regulated genes that have an ortholog in a fly-oriented way. Numbers on the axes outside the triangle indicate the number of fly genes that map an ortholog in zebrafish and mouse. Numbers at vertices indicate the number of up-regulated orthologs in regeneration and in each species. The set of up-regulated genes shared between the three species is highlighted, and the bar plot shows GO term enrichment for them. (*C*) Up-regulated and shared TFs acting as effectors of regeneration signaling pathways. (*D*) Schematic representation of the experimental design used to score the capacity to regenerate after the depletion of a gene by RNAi and the induction of cell death (*top*). Adult wings showing the predominant phenotype observed in each condition (*middle*). The region where the RNAi was expressed is highlighted in yellow and the apoptotic region in orange. The box plot shows the average area of adult wings obtained after the expression of cell death, the RNAi, or the combination of both (*bottom*). Each dot represents one wing; wild-type pattern (gray) and aberrant pattern (orange). (*E*) Classification of DRREs identified in fly and zebrafish as novel eDRREs, reused eDRREs, and iDRREs based on co-option from embryo stages.

First, we identified the fly protein-coding genes that have orthologs in at least one of these two species (7458 genes; 54% of all fly genes). We found that fly genes that are up-regulated in the early stages after injury have more vertebrate orthologs than down-regulated genes or genes overall; at the early stage, 65% of up-regulated genes have vertebrate orthologs, whereas only 35% of down-regulated genes have vertebrate orthologs (Fig. 6A; Supplemental Fig. S17).

Next, we analyzed RNA-seq data produced recently during heart regeneration in zebrafish (Goldman et al. 2017) and liver regeneration in mouse (Sun et al. 2016). We identified the genes that were up-regulated after injury in zebrafish and in mouse using the same bioinformatics protocol that we used in fly. We obtained 2476 fly genes with at least one ortholog that is up-regulated in zebrafish regeneration and 2347 fly genes with at least one ortholog that is up-regulated in mouse regeneration. This compares to 2162 genes up-regulated in fly regeneration that have vertebrate orthologs (Fig. 6B). Fly regeneration genes are not more likely to have regeneration orthologs in mouse and zebrafish than expected by chance (Supplemental Fig. S17). When we compared the three gene sets, we found 268 genes shared by all species. In this set, we found enrichment of GO terms similar to those obtained when considering fly regeneration alone (Fig. 1F), with the highest enrichment in terms related to DNA binding and TF activity (Fig. 6B).

Of the shared genes, we found 21 TFs, seven of which are effectors of signaling pathways involved in regeneration (Fig. 6C; Supplemental Table S6). From these, we selected *Dif* (up-regulated at the early and late time points), *Lilli* (up-regulated at the early time point), and *Stat92E* (up-regulated at the mid time point) (Supplemental Figs. S5, S6) to study their requirement in fly regeneration. We used the double transcriptional transactivator system consisting of *sal^E/Pv^-LHG lexO-rpr* to induce apoptosis, combined with the *UAS/Gal4* and *Gal80*^*ts*^ systems to drive the expression of the RNAi against the TFs in the anterior domain (*cubitus interruptus*, *ci-Gal4*) of the wing disc (Santabárbara-Ruiz et al. 2015). As previously reported (Bergantiños et al. 2010a; Repiso et al. 2013; Santabárbara-Ruiz et al. 2015), we found that the induction of cell death resulted in a properly regenerated adult wing (Fig. 6D). The expression of each RNAi without the induction of cell death had a minor effect on the adult wings, characterized by a slight decrease in the area and by 10% of wings having a slight patterning defect. However, the combination of each individual RNAi with genetic ablation resulted in a major reduction of wing area as well as an increase in the number of wings with aberrant vein patterning (100%, 70%, and 90% for *Dif*, *Lilli*, and *Stat92E*, respectively). These results demonstrate that these TFs are not only up-regulated but are also required for proper regeneration.

Next, we examined to what extent the pattern of emergence and reusage of DRREs that we found in fly is also present in other systems. We analyzed the only available data on open chromatin in regeneration, which is the histone profiling of H3.3 from the zebrafish heart regeneration experiment cited above (Goldman et al. 2017). We followed the same bioinformatics procedure that we used in fly to obtain DRREs in this model (Methods). In agreement with the published results (Goldman et al. 2017), we found that the majority of DRREs in zebrafish are emerging, in contrast to the case of fly, where the great majority of DRREs are increasing (Fig. 6E). To study the possible reusage of eDRREs, we analyzed open chromatin data obtained during zebrafish embryonic development (Gehrke et al. 2015). As observed in the fly, we found that a fraction of open regions classified as emerging had already been identified as enhancers in embryos—reused eDRRE (47% of all DRREs) (Fig. 6E; Supplemental Fig. S17).

## Discussion

In this work, we have identified the genes and regulatory elements involved in regeneration of fly imaginal discs. Our results indicate that major transcriptional changes occur during the first steps of the recovery process, and that a number of up-regulated genes, including key TFs, also act during regeneration in other animals. These could therefore constitute the regenerative core of metazoans. In addition, we have distinguished several classes of enhancers that operate during regeneration.

Early signals that initiate regeneration in different species involve calcium waves and the production of reactive oxygen species (ROS) (Razzell et al. 2013; Santabárbara-Ruiz et al. 2015; Niethammer 2016; Hariharan and Serras 2017). This early burst of ROS activates the JNK and p38 MAPK pathways, which in turn activate the Jak-STAT pathway (Gauron et al. 2013; Santabárbara-Ruiz et al. 2015; Fogarty et al. 2016). Similar to the case of heart and liver regeneration, expression of several elements from the same pathways (i.e., *Jra*, *p38a,* and *upd1*) is induced after damage of imaginal discs, which indicates that more sustained activity is probably necessary for organ growth.

Around 34% of up-regulated genes are located in genomic clusters. These clusters are enriched in genes operating in signaling pathways, with some clusters containing members from different pathways. For example, one cluster of up-regulated genes includes, among others, a member of the Jak-STAT pathway (*dome*), the Hippo pathway (*mer*), and the MAPK pathway (*cdc42*). Such distribution could represent an efficient regulatory strategy, because many genes required for early repair could be turned on at once, in bulk, by cluster coregulation. Our results obtained using conformation capture experiments suggest global cluster regulation by a well-positioned enhancer element; however, further experiments are necessary to examine whether this is a generalized phenomenon.

The observation that organs and tissues of a single species have different regenerative capabilities indicates that the capacity to regenerate does not depend only on the genome sequence, but also on genome activity. Here, we have identified DRREs as specific regions in the genome that become activated after cell death and are highly conserved through different *Drosophila* species. DRREs could have been positively selected for, thereby allowing fly survival after environmental aggressions that may alter cell homeostasis and activate a damage response.

We have classified DRREs into three different types. Some DRREs are in regions that are already open in the wing imaginal disc, but that become more accessible during regeneration, indicating a fine-tuning mechanism, as occurs with the WNT damage enhancer (Harris et al. 2016). Some others are co-opted from other developmental stages or tissues and are reused in regeneration. For regeneration to take place, cells have to proliferate and change their fate to replace the lost tissue; it is known that the pathways that drive proliferation following tissue damage are the same as those that regulate growth during development (Hariharan and Serras 2017; Chen and Poss 2017). The existence of elements used during development and reused in injury was previously proposed for zebrafish regeneration (Chen and Poss 2017), and our work confirms that accessible and active regions can participate in development as well as in regeneration both in flies and zebrafish. Finally, a fraction of DRREs correspond to novel enhancers that act exclusively in damaged tissue. Although this last category could, in theory, represent genuine regeneration enhancers, it seems likely that the complete regenerative response requires the combinatorial effect of all the classes of enhancers. Altogether, our results suggest a gene regulatory program triggered by different types of DRREs acting either on individual genes or on clusters of coregulated genes.

DRREs contain conserved binding motifs for TFs that are downstream from signaling pathways. These TFs (i.e., Stat92E, Sd, and Myb) are not only expressed but up-regulated and required in the regenerating of organs in fly, zebrafish, and mouse (Sun et al. 2016; Goldman et al. 2017). The existence of regions capable of responding to damage or stress that become active in the presence of specific TFs and are shared across organ regeneration in different species may have profound implications for our understanding of tissue regeneration. Specific regions of the genome that have the potential to act as enhancers can be activated in one place or another depending on the needs of the cell and the combinatorial action of TFs that are present at a particular moment in the cell. Ectopic activation of regeneration enhancers could potentially be used to stimulate the regenerative capacity of organs and tissues that, in principle, are not able to regenerate.

## Methods

### *Drosophila* strains

The *Drosophila melanogaster* stocks used were *w*^*1118*^ (Bl 5905), *UAS-rpr* (Wing et al. 1998), *LexO-rpr* (Santabárbara-Ruiz et al. 2015), *salm-Gal4* (Bergantiños et al. 2010a), *sal^E/Pv^-LHG* (Santabárbara-Ruiz et al. 2015), *ci-Gal4* (Martín and Morata 2006), and *tub-Gal80^ts^* (McGuire et al. 2003). For enhancer validations, we used FlyLight-Janelia lines obtained from Bloomington Stock Centre and VT lines obtained from the Vienna Drosophila Resource Center (VDRC) (http://stockcenter.vdrc.at), as well as *UAS-mCD8GFP* (Bl. 32186). RNAi lines were also obtained from VDRC. A detailed list of the stocks used is provided in Supplemental Methods.

### Induction of cell death and physical injury

Cell death in the wing imaginal disc was induced as previously described (Bergantiños et al. 2010a; Repiso et al. 2013). Expression of the proapoptotic genes *rpr* was driven using *salm-Gal4* in combination with the thermosensitive repressor *tub-Gal80^ts^.* Induction was performed for 16 h in all the experiments. Control samples without *rpr* expression were always treated in parallel. For enhancer validation and RNAi analysis, we used the *sal^E/Pv^-LHG* and *LexO-rpr* strains for genetic ablation, using the same design as for Gal4/UAS (Santabárbara-Ruiz et al. 2015). For analysis of physical injury, discs were cut and cultured for 8 h at 25°C as previously described (Santabárbara-Ruiz et al. 2015). A detailed list of the genotypes used is provided in Supplemental Methods.

### Immunohistochemistry

For apoptotic cell detection, we used the TUNEL assay with fluorescently labeled dUTP ChromaTide BODIPY FL-14-dUTP (Life Technologies), incorporated using terminal deoxynucleotidyl transferase (Roche). As patterning markers, we used DSRF (Active Motive, 39093) and Phalloidin-Rhodamine (Life Technologies, R415). For mitosis detection, we used H3P histone H3 (phospho S10) (Abcam ab47297). Nuclei were stained using NucRed (Life Technologies) for in vivo imaging and TO-PRO-3 (Life Technologies) for fixed tissues. Images were captured using a Leica SPE confocal microscope and processed and treated with ImageJ and Adobe Photoshop 7.0 software.

### RNA-seq library preparation and sequencing

We used 40 wing discs for each genotype (regeneration and control) and time point (early, mid, and late). Two biological replicates of each sample were performed. RNA was extracted with ZR RNA microprep and RNA clean and concentrator kits (Zymo Research). Five micrograms of total RNA were used for reverse transcription, and cDNAs were subjected to Illumina TruSeq library preparation. All libraries were sequenced on an Illumina NextSeq 500, according to the manufacturer's instruction. Sequencing was performed by Sandor Life Sciences.

### ATAC-seq library preparation and sequencing

We used 10 wing discs for each genotype (regeneration and control) and time point (early, mid, and late) as well as the third instar larva (L3). Two biological replicates of each sample were performed as previously described (Davie et al. 2015; Gehrke et al. 2015) with some modifications. Briefly, the samples were lysed in Lysis Buffer (10 mM Tris-HCl at pH 7.4, 10 mM NaCl, 3 mM MgCl_2_, 0.1% NP-40) by gently pipetting. Lysates were centrifuged for 10 min at 500*g* to isolate the nuclei. Nuclei were resuspended and incubated for 30 min at 37°C in transposition reaction mix (Illumina). Immediately after the transposition reaction, the samples were purified using a Qiagen MinElute Kit and eluted in Elution Buffer (10 mM Tris buffer at pH 8). For library preparation, we amplified the transposed DNA fragments by running a conventional PCR (5 min at 72°C, 2.5 min at 95°C, the thermocycling: 13 cycles of 20 sec at 98°C, 15 sec at 63°C, and 1 min at 72°C) with Nextera barcoded primers. Libraries were purified using a Qiagen PCR CleanUP Kit and eluted in Elution Buffer. All the libraries were sequenced on an Illumina HiSeq 2500 according to the manufacturer's instruction. Sequencing was performed at the Centre Nacional Anàlisi Genòmica (CNAG-CRG) sequencing facility in Barcelona, Spain.

### RNA-seq data processing and analysis

Data were processed using grape-nf (https://github.com/guigolab/grape-nf). RNA-seq reads were aligned to the fly genome (dm6) using STAR 2.4.0j software (Dobin et al. 2013) with up to four mismatches per paired alignment using the FlyBase genome annotation r6.05. Only alignments for reads mapping to 10 or fewer loci were reported. Gene and transcript FPKMs were quantified using RSEM (Li and Dewey 2011). Genes showing at least a 1.7-fold change difference in expression levels between control and regeneration at each time point were considered DE. Plots were made using d3js (https://d3js.org/), ggplot2 (Wickham 2009), and R scripts (https://github.com/abreschi/Rscripts).

We used the DAVID (Huang et al. 2009a,b) web tool to identify GO terms. For time-course analysis of molecular function terms, we used reviGO (Supek et al. 2011) to compute a network based on semantic terms, term enrichment, and gene number for each time; we used Cytoscape (Shannon et al. 2003) to merge and visualize all time points. We used KEGG Mapper (Kanehisa and Goto 2000; Kanehisa et al. 2016, 2017) to map up-regulated genes in fly pathways.

TF annotation was obtained from FlyFactorSurvey (http://mccb.umassmed.edu/ffs).

Chromosomal clusters were identified for early, mid, and late up-regulated and down-regulated protein-coding genes using CROC with default parameters: --min_genes 3 --window 30000 --offset 10000 --pva 0.05 --fdr Benjamini and Hochberg (Pignatelli et al. 2009). The expression profile of genes inside clusters in regeneration samples during the three time points was analyzed as follows: Genes for which maximal expression divided by minimal expression was greater than two FPKM were considered variable; the others were classified based on the average expression at the three time points (highly expressed for average expression greater than 30 FPKM; moderately expressed for average expression greater than five FPKM and smaller than or equal to 30; lowly expressed for average expression greater than one FPKM and smaller than or equal to five; and silenced for average expression smaller than or equal to one FPKM). To assess coregulation of genes in the same cluster, we computed the Pearson coefficient of correlation for every protein-coding gene pair over time, using the R script gene.pair.correlation.R with parameters --log --pseudocounts 0.01 (https://github.com/abreschi/Rscripts). Cluster hotspots were also identified using CROC on chromosomal clusters. For this, a window of 1,000,000 bp was defined, and no *P*-value or multiple test correction was required.

### ATAC-seq data processing and analysis

Reads were continuously mapped to the fly genome (dm6) using STAR 2.4.0j software (Dobin et al. 2013). Only uniquely aligned reads to canonical chromosomes were selected. To generate the nucleosome position data, reads shorter than 100 bp were considered NF, and reads between 180 and 247 bp were considered to be MNs (Buenrostro et al. 2015). Peaks were called using the paired-end mode of MACS2 software (Zhang et al. 2008), and signal profiles were normalized by the total number of sequenced reads. Concordant peaks (i.e., those called in both replicates) of all the samples were merged to define a set of consensus regions using BEDOPS v. 2.4.14 (Neph et al. 2012).

To identify differentially accessible regions, we pairwise compared peaks called in control and regeneration at each time point. We analyzed the presence and absence of peaks or peak summits showing at least a 1.5-fold change difference in height when called in both conditions. The peak height of each sample was defined using bwtool summary v. 1.0 (Pohl and Beato 2014).

We assigned a unique genomic annotation for each peak using the following order: CP (±100 bp from the TSS); FI (region between the first and second projected exons, i.e., merged exons of all the annotated transcripts of a gene); proximal (±2 kb from the TSS); distal (more than ±2 kb from the TSS).

### ChIP-seq library preparation and sequencing

We isolated 100 wing discs per sample (early control and regeneration). The discs were fixed, pooled in 700 µL of sonication buffer (10 mM Tris-HCl at pH 8.0, 2 mM EDTA, and 1 mM EGTA) and processed as described elsewhere (Pérez-Lluch et al. 2011). Immunoprecipitations were performed in RIPA buffer using 1 µg of the corresponding antibody. Immunocomplexes were recovered by incubation with Invitrogen Protein A magnetic beads for 2 h. The beads were washed three times in RIPA, once in lithium chloride buffer, and twice in TE buffer. Afterward, RNase treatment was performed and the samples were de-crosslinked at 65°C overnight by adding Proteinase K. Samples were purified with a Qiagen MinElute Kit and eluted in Gibco water. Library preparation and sequencing using HiSeq 2000 was carried out at CRG Genomics Unit (Barcelona, Spain).

ChIP-qPCR analysis was performed following the same protocol. ChIP eluates and input (10%) were assayed by real-time PCR with SYBR Master Mix (Roche) (the primers are listed in Supplemental Methods). The ΔΔCt method was used to normalize the data. Both samples were normalized against the input. Average standard error of the mean of two biological replicates was computed for each based on three technical replicates by the ΔΔCt method. ChIP enrichment is shown as fold enrichment between regeneration and control.

The antibodies used for ChIP assays were H3K4me1 (Diagenode, CS-037-100), H3K27ac (Abcam, ab4729), Pol II-8WG16 (Abcam, ab817), H3K27me3 (Upstate-Millipore, 07-449), and Pol II phospho ser5 (Abcam, ab5131).

### ChIP-seq data processing and analysis

Data were processed using chip-nf pipeline (https://github.com/guigolab/chip-nf). Reads were continuously mapped to the fly genome (dm6) with up to two mismatches using GEM mapper (Marco-Sola et al. 2012). Only alignments for reads mapping to 10 or fewer loci were reported. Duplicated reads were removed using Picard (http://broadinstitute.github.io/picard/). Fragment length was estimated using SPP (Kharchenko et al. 2008; Landt et al. 2012). Peak calling was performed using MACS2 (Zhang et al. 2008). Signal profiles were quantile normalized using R package preprocessCore (Bolstad et al. 2003). The quality check was based on the signal level of H3K27ac, H3K4me1, and Pol II at the TSS of modENCODE stable and silent genes (Graveley et al. 2011). We computed the coefficient of variation of gene expression for 12 developmental time points and selected 1000 stable genes (lowest values of the coefficient of variation) and 1000 silent genes from this same data set. Active enhancer features included H3K4me1, H3K27ac, and Pol II. The classification for each feature was as follows: (1) marked, presence of a peak in regeneration samples in a window of 500 bp up/downstream from the ATAC-seq peak; (2) higher signal, higher average signal in regeneration compared to control samples at the same time point; and (3) not marked, none of the previously described cases. Final active enhancer classification: (1) presence, at least one of the features was marked or with higher signal; or (2) absence, all features were classified as not marked (Supplemental Table S3).

### Enhancer validation using reporter lines

We crossed the *UAS-mCD8GFP* line with the FlyLight-Janelia and VT lines containing sequences of eDRREs and iDRREs, as well as negative controls cloned upstream of a Gal4 (Pfeiffer et al. 2008), and tested them after genetic ablation and physical injury. A detailed list of fly lines and genotypes is provided in Supplemental Methods.

### Chromosome conformation capture followed by real-time PCR (3C-qPCR)

We developed a 3C protocol for wing disc following previously described 3C procedures for *Drosophila* (Li 2016). We used 300 wing discs for each condition (early control and early regeneration), and we performed two replicates of each experiment. Rounds of 50 larvae (100 discs each) were turned and fixed in 37% formaldehyde in 1× PBS for 15 min at 25°C. Fixation was quenched with glycine (0.125 M) and cooled down on ice for 5 min. The larvae were resuspended in 1× PBS, and the discs were dissected. All the discs were pooled together and spun down, then lysated in Lysis Buffer (10 mM Tris-HCl at pH 7.4, 10 mM NaCl, 3 mM MgCl_2_, 0.1% NP-40) by gently pipetting for 10 min. Nuclei were pelleted by centrifugation at 600*g* and 4°C for 10 min, then washed for 5 min in 1× Restriction Enzyme Buffer (PstI). Nuclei were centrifuged again, resuspended and incubated for 1 h at 37°C in Nuclei Lysis Buffer (1× Restriction Enzyme Buffer, SDS). Triton X-100 was added (final concentration, 2%) for 1 h more. PstI (Promega) was added to the sample and incubated overnight at 37°C; afterward, SDS was added for 1 h. Following the addition of ligation buffer and Triton X-100, the samples were incubated for 1 h at 37°C. The temperature was lowered by incubation on ice for 5 min and then ATP and T4 ligase (Roche) were added. The ligation reaction lasted for 4 h at 16°C and for 1 h at 25°C. After ligation, the samples were de-crosslinked at 65°C overnight by adding Proteinase K. Immediately afterward, RNase treatment was performed. The samples were purified with a Qiagen MinElute Kit and eluted in Gibco water. 3C eluates were assayed by real-time PCR with SYBR Master Mix (Roche) (the primers are listed in Supplemental Methods). The ΔΔCt method was used to normalize the data. 3C interaction enrichment is shown as fold enrichment between regeneration and control. Both samples were normalized against a known interaction in *Drosophila* (Bieli et al. 2015). The average standard error of the mean of two biological replicates was calculated for each sample based on three technical replicates by the ΔΔCt method.

### Genome-wide comparative analysis

To characterize the predicted enhancers, raw reads of available ChIPs of H3 and H3K27me3 of third instar larvae imaginal discs of *D. melanogaster* were obtained from NCBI GEO database GSE56551 (Pérez-Lluch et al. 2015) and GSE74080 (Loubière et al. 2016), and were processed using chip-nf pipeline (https://github.com/guigolab/chip-nf). Reads were continuously mapped to the fly genome (dm6) with up to two mismatches using GEM mapper (Marco-Sola et al. 2012). Only alignments for reads mapping to 10 or fewer loci were reported. Fragment length was estimated using SPP (Kharchenko et al. 2008; Landt et al. 2012). Peak calling was performed using MACS2 (Zhang et al. 2008).

For reusage analysis, FAIRE data of fly developmental stages and tissues were also obtained from NCBI GEO database GSE38727 (McKay and Lieb 2013). Peak coordinates were converted from dm3 to dm6 using the liftOver tool from UCSC Genome Browser (Tyner et al. 2017). DRREs overlapping FAIRE open regions in developmental stages other than L3, or in tissues other than the wing imaginal disc, were considered to be reused. This overlap was computed using BEDTools intersectBed v2.17.0 (Quinlan and Hall 2010).

DRRE conservation was studied using the dm6 27-way multiple alignment (23 *Drosophila* sequences, house fly, *Anopheles* mosquito, honey bee, and red flour beetle) and the phastCons measurement of evolutionary conservation from the UCSC Genome Browser (Tyner et al. 2017). The bwtool was used to intersect peaks with the conservation track (Pohl and Beato 2014). Predicted enhancers were also compared with STARR-seq data on genome-wide enhancer activity profiles for five *Drosophila* species: *D. ananassae*, *D. melanogaster*, *D. pseudoobscura*, *D. yakuba*, and *D. willistoni* from the NCBI GEO database (GSE48251, GSE40739) (Arnold et al. 2014).

### Motif enrichment analysis

We used iCIS target (Herrmann et al. 2012) with default parameters to obtain the enriched motifs in each enhancer type. Only TFs expressed in the RNA-seq (>1 FPKM) were considered a hit. Redundant hits were manually removed.

### Conservation of the regeneration regulatory logic

Fly gene identifiers were mapped to zebrafish and mouse orthologs using Ensembl79 (http://mar2015.archive.ensembl.org) (Yates et al. 2016). Genes mapping to one or more orthologs in zebrafish or mouse were analyzed in a fly-oriented manner.

To identify regeneration core genes, we selected fly up-regulated genes (early and/or mid) mapping to at least one up-regulated ortholog in the data sets detailed in what follows. Transcriptional profiling of regeneration in zebrafish heart and in mouse liver were obtained from NCBI GEO database: GSE81865 (Goldman et al. 2017) and GSE76926 (Sun et al. 2016). We identified higher-expressed genes in regeneration for each species (at least a 1.5-fold change difference between injured and uninjured expression levels). The set of genes up-regulated in fly, in zebrafish, and in mouse regeneration data were used to identify the regenerative core genes.

A genome-wide map of histone variant H3.3 occupancy in zebrafish cardiomyocytes undergoing regeneration (same experimental conditions as the RNA-seq zebrafish heart data) was compared to an uninjured sample (NCBI GEO database GSE81893) (Goldman et al. 2017). Concordant peaks (i.e., peaks called in both replicates) were classified as *emerging* (eDRRE: exclusively called in regeneration) or *increasing* (iDRRE: called both in uninjured and injured samples, and at least 1.5-fold higher in samples undergoing regeneration). Peaks were classified based on nonoverlapping regions of genomic location: CP (0.5 kb up/downstream from the TSS); first intron (region between the first and second projected exons, i.e., merged exons of all the annotated transcripts of a gene); proximal (±2 kb from the TSS); distal (±more than 2 kb from the TSS), based on Ensembl release 89 of zebrafish (GRCz10) (Yates et al. 2016). DRREs were compared to ATAC-seq data from 24 h post-fertilization (hpf) zebrafish embryo (NCBI GEO database GSE61065) (Gehrke et al. 2015). Raw zebrafish ATAC-seq data were mapped to the GRCz10 assembly and processed as described for the fly ATAC-seq NF fraction presented herein. Reusage analysis was based on the overlap between zebrafish DRRE with open regions in embryo, as for fly.

### Test for regenerated adult wings

To test the capacity to regenerate, we analyzed adult wings emerged from flies in which cell death had been induced using the *LHG/lexO* system, and genes were depleted by RNAi using the *UAS/Gal4* system. We activated both systems for 16 h on the eighth day after egg laying. Adult flies were fixed in glycerol:ethanol (1:2) for 24 h. Wings were mounted on 6:5 lactic acid:ethanol, and both were analyzed and imaged under a microscope. A detailed list of fly lines and genotypes is provided in Supplemental Methods.

### UCSC track data hub

Processed data from this study is available for visualization at the UCSC Genome Browser (Tyner et al. 2017): (1) signal files for all data types; (2) location of genomic clusters; (3) nucleosome-free peaks of ATAC-seq (concordant peaks between replicates); and (4) ChIP-seq peaks.

## Data access

RNA-seq, ATAC-seq, and ChIP-seq raw and processed data from this study have been submitted to the NCBI Gene Expression Omnibus (GEO; http://www.ncbi.nlm.nih.gov/geo/) under accession number GSE102841. All the data is also available in the UCSC track data hub (https://public-docs.crg.es/rguigo/Papers/2018_vizcaya-klein_regeneration/hub.txt).

## Supplementary Material

Supplemental Material
