# Supplementary material for: Damage-responsive elements in Drosophila regeneration
Source: Genome Res. 2018 Dec;28(12):1852–66. doi: 10.1101/gr.233098.117 (PMC6280756; doi:10.1101/gr.233098.117)
Supplement: Supplemental Material [file supp_28_12_1852__index.html]

Damage-responsive elements in Drosophila regeneration — Supplemental Material 

# Damage-responsive elements in *Drosophila* regeneration

## Supplemental Material

- Supplemental\_Figures.pdf
- Supplemental\_Methods.pdf
- Supplemental\_Table\_Legends.pdf
- Supplemental\_Table\_S1.xlsx
- Supplemental\_Table\_S2.xlsx
- Supplemental\_Table\_S3.xlsx
- Supplemental\_Table\_S4.xlsx
- Supplemental\_Table\_S5.xlsx
- Supplemental\_Table\_S6.pdf
