# Supplementary material for: Damage-responsive elements in Drosophila regeneration
Source: Genome Res. 2018 Dec;28(12):1852–66. doi: 10.1101/gr.233098.117 (PMC6280756; doi:10.1101/gr.233098.117)
Supplement: Supplemental Material [file supp_gr.233098.117_Supplemental_Figures.pdf]

SUPPLEMENTAL FIGURES FOR

“Damage responsive elements in *Drosophila* regeneration”

Elena Vizcaya-Molina, Cecilia C. Klein, Florenci Serras, Rakesh Mishra, Roderic Guigó and Montserrat Corominas

TABLE OF CONTENTS

Supplemental Fig. S1 - RNA-seq analysis workflow

Supplemental Fig. S2 - ATAC-seq analysis workflow

Supplemental Fig. S3 - Statistics and replicate analysis of RNA-seq

Supplemental Fig. S4 - Statistics and replicate analysis of ATAC-seq

Supplemental Fig. S5 - Expression profiles of upregulated transcription factors

Supplemental Fig. S6 - Pathway enrichment in upregulated genes

Supplemental Fig. S7 - Features of genomic clusters

Supplemental Fig. S8 - Gene Ontology of differentially expressed genes

Supplemental Fig. S9 - Statistics and replicate analysis of third instar larval ATAC-seq

Supplemental Fig. S10 - Accessible chromatin landscape after cell death induction

Supplemental Fig. S11 - Statistics and analysis of ChIP-seq

Supplemental Fig. S12 - Chromatin features of DRREs

Supplemental Fig. S13 - Validation of the activity of DRREs after damage

Supplemental Fig. S14 - Tissue usage of DRREs

Supplemental Fig. S15 - Reusage and conservation of DRREs

Supplemental Fig. S16 - Conservation of DRREs

Supplemental Fig. S17 - Homology of genes implicated in fly regeneration

Supplemental Figures References

Supplemental Figure S1

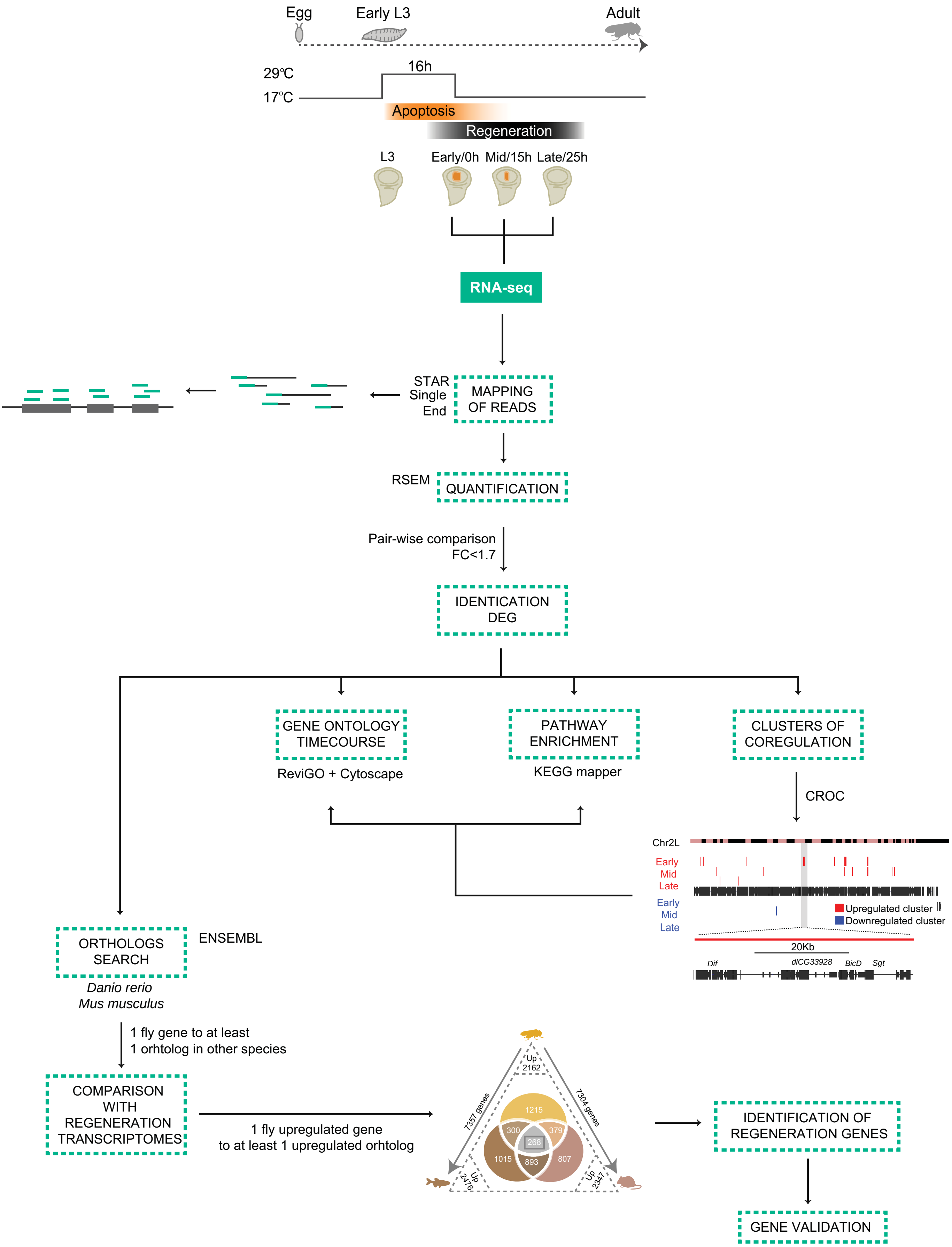

RNA-seq analysis workflow. Workflow of the logic and the analysis carried out.

Supplemental Figure S2

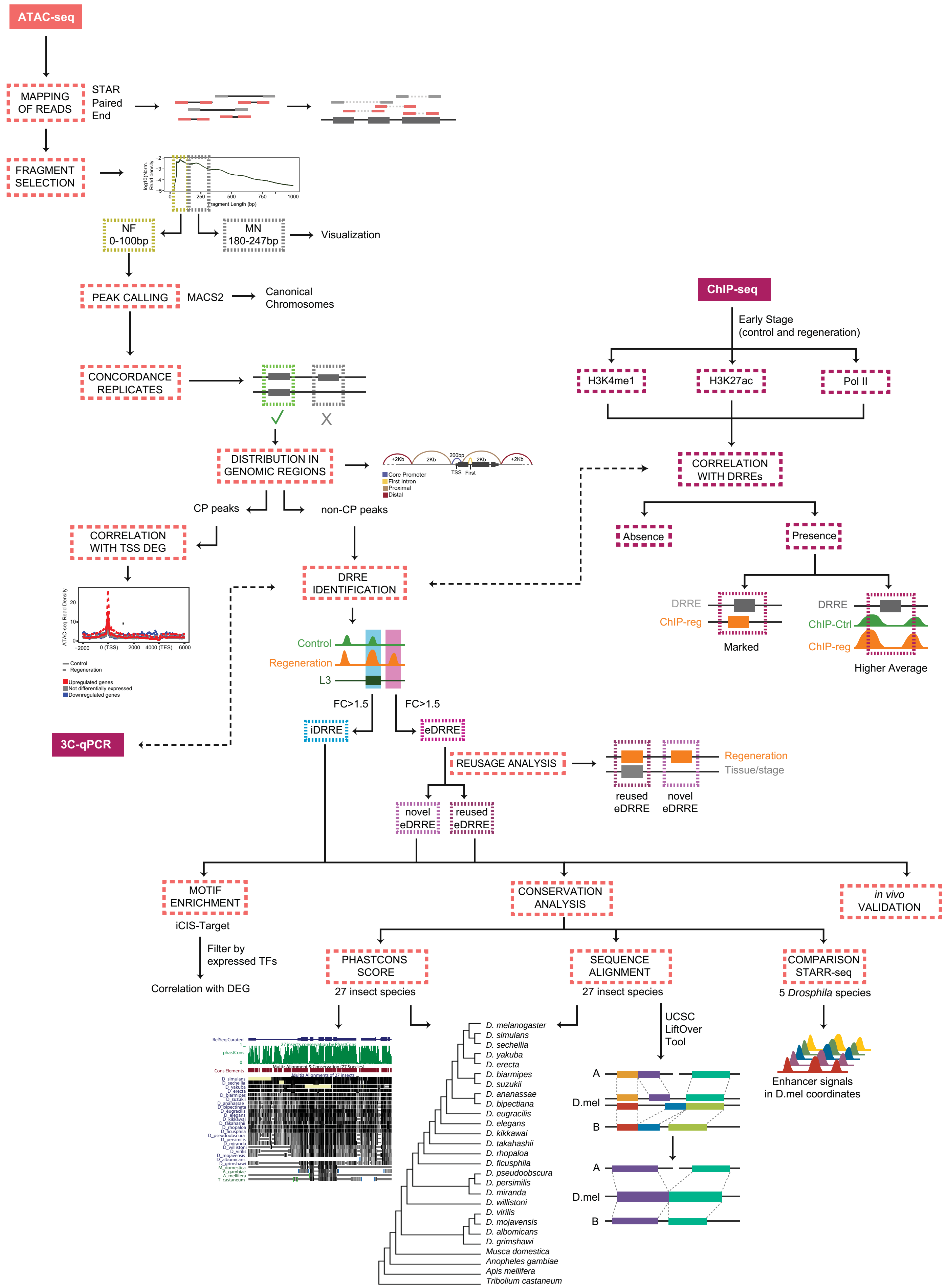

ATAC-seq analysis workflow. Workflow of the logic and the analysis carried out.

Supplemental Figure S3

A

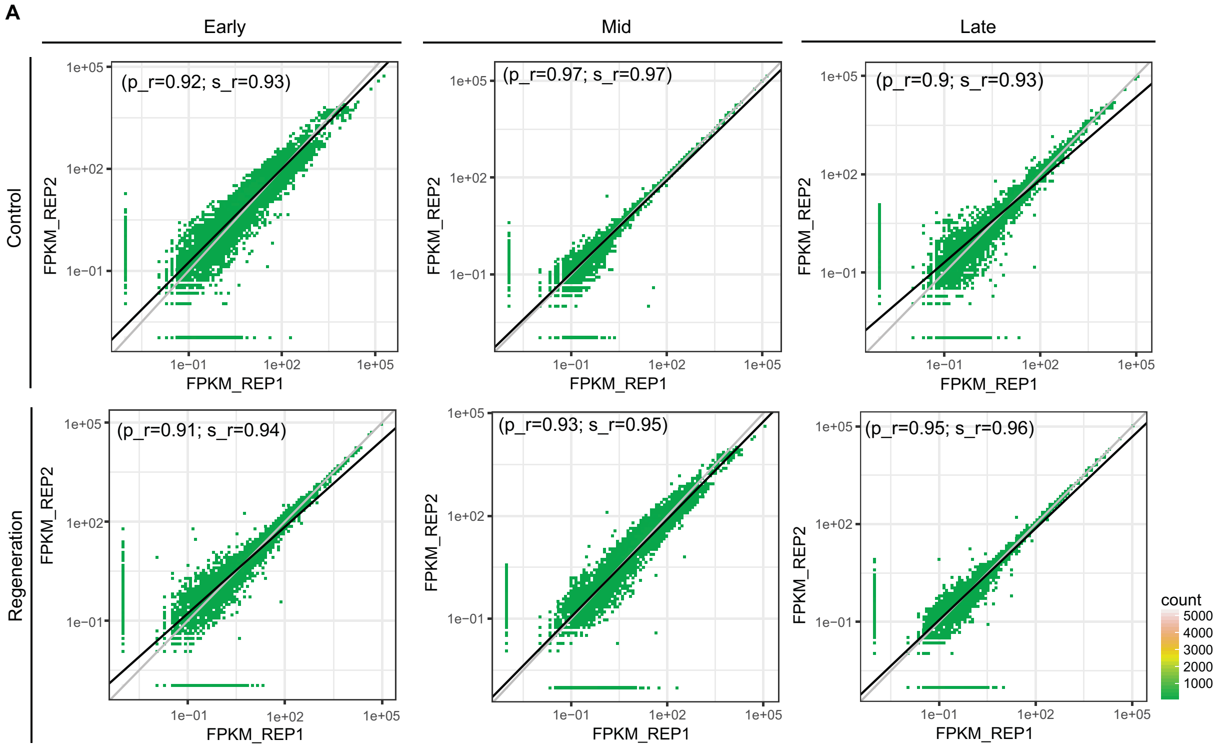

B

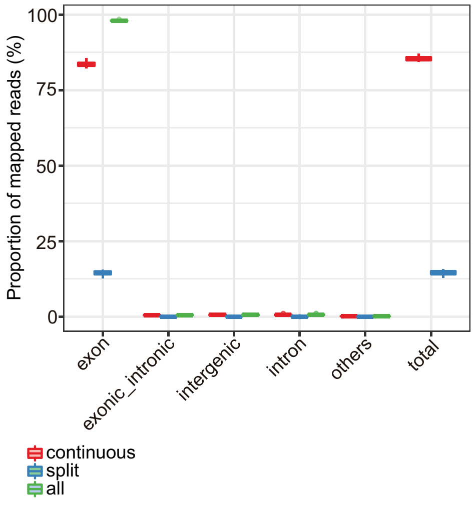

C

Exp-Id	nb_map_read	nb_uniq_read	prop_map_read	prop_uniq_read
ER-ctrl_rep1	19323620	19065888	83.09	98.67
ER-ctrl_rep2	36442984	35913484	92.95	98.55
ER-reg_rep1	17166784	16797954	83.74	97.85
ER-reg_rep2	19738296	19394714	88.38	98.26
MR-ctrl_rep1	52155568	51379660	95.71	98.51
MR-ctrl_rep2	68513640	67479508	96.43	98.49
MR-reg_rep1	18119846	17824258	89.62	98.37
MR-reg_rep2	18338768	17957018	85.59	97.92
LR-ctrl_rep1	18198962	17913950	93.17	98.43
LR-ctrl_rep2	38587464	37937572	91.59	98.32
LR-reg_rep1	19571362	19181440	85.10	98.01
LR-reg_rep2	26791616	26266120	87.07	98.04

D

	Early			Mid			Late		
	Up	NDE	Down	Up	NDE	Down	Up	NDE	Down
mRNA	1997	11763	160	1631	12095	194	617	12749	554
ncRNA	99	2278	56	52	2311	70	105	2287	70
pseudogene	14	287	7	14	288	6	16	288	4
rRNA	15	126	6	12	130	5	8	135	4
tRNA	0	313	0	0	313	0	0	313	0
Total	2125	14803	230	1709	15173	276	747	15808	603

E

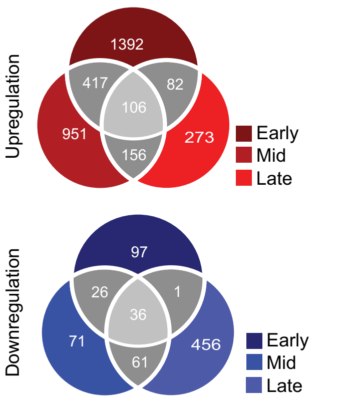

Statistics and replicate analysis of RNA-seq. RNA-seq was performed from two independent biological replicates from each time point and condition. (A) Scatter plots showing high correlation of gene expression levels between replicates (Pearson and Spearman correlation coefficients higher than 0.9, denoted by p_r and s_r, respectively). (B) Mapped genomic reads were classified as: exonic if reads map entirely within exons, exonic-intronic if reads map both in exons and introns, intergenic if reads map outside genes and intronic if reads map entirely within a gene but not within annotated exons. Split reads were reads mapping to splice junctions. (C) RNA-seq mapping statistics. Number and proportion of mapped reads and unique mapped reads are shown. Most reads (98%) map to the exons. (D) Number of differentially expressed genes at all time points. Gene types are specified. (E) Venn diagram showing the intersection of DE genes in the three time points.

Supplemental Figure S4

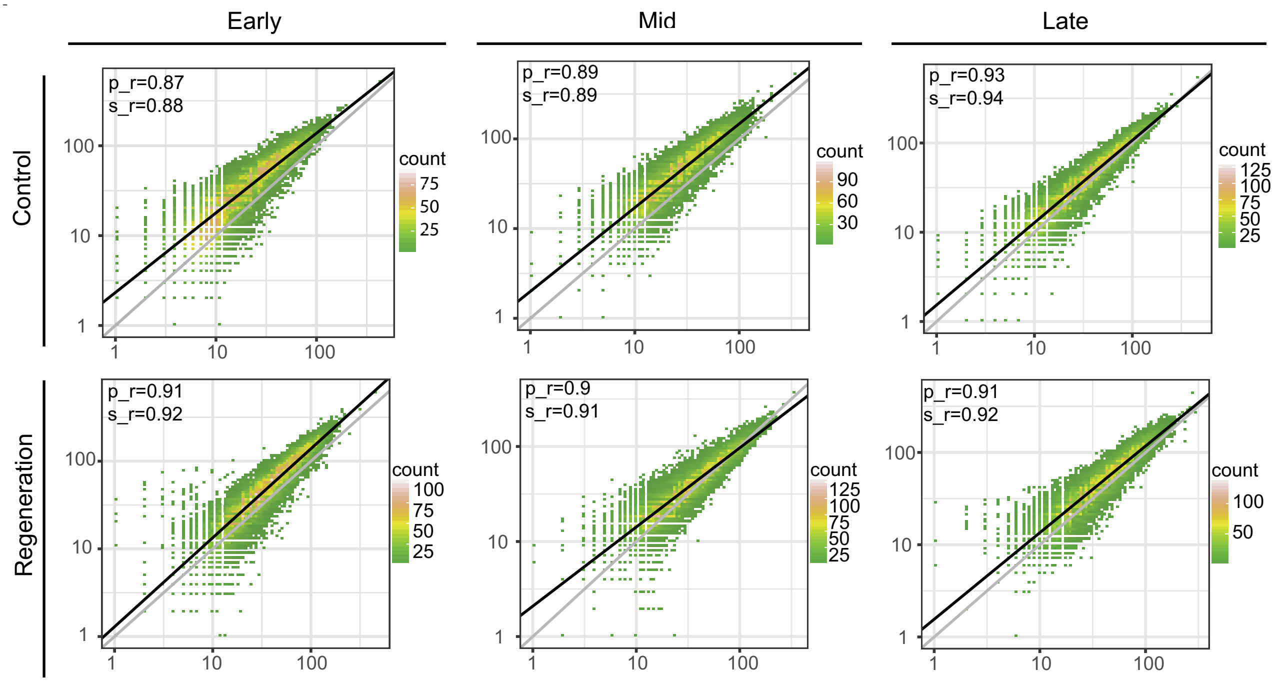

B

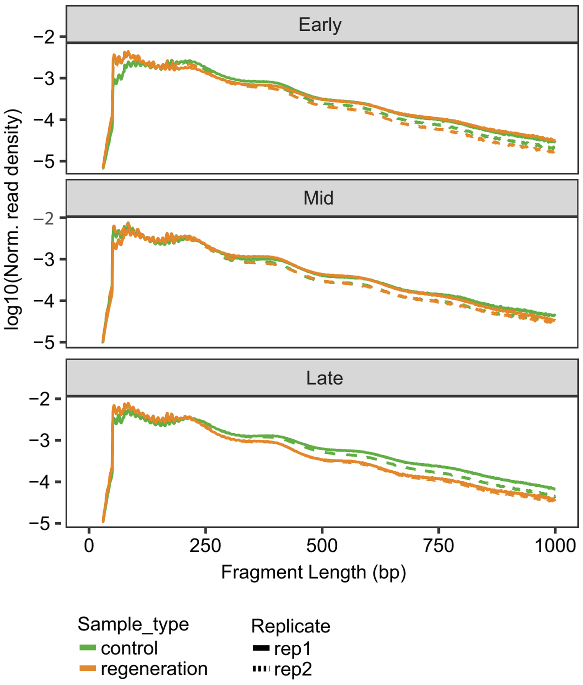

C

labExpId	total	uniq	prop_uniq
ER-ctrl_NF_rep1	5341626	5199086	97.3
ER-ctrl_NF_rep2	10242146	9975064	97.3
ER-reg_NF_rep1	10775260	10466302	97.1
ER-reg_NF_rep2	11857222	11559318	97.4
MR-ctrl_NF_rep1	8261020	7960502	96.3
MR-ctrl_NF_rep2	12779268	12368228	96.7
MR-reg_NF_rep1	9441288	9178988	97.2
MR-reg_NF_rep2	12362538	11997774	97.0
LR-ctrl_NF_rep1	9936622	9672454	97.3
LR-ctrl_NF_rep2	11242928	10976686	97.6
LR-reg_NF_rep1	10745466	10382710	96.6
LR-reg_NF_rep2	13657716	13253602	97.0

D

labExpId	total	uniq	prop_uniq
ER-ctrl_MN_rep1	10876938	110526732	96.7
ER-ctrl_MN_rep2	10286054	998006	97.0
ER-reg_MN_rep1	8023184	766414	96.7
ER-reg_MN_rep2	10384794	10065668	96.9
MR-ctrl_MN_rep1	7126620	6811380	95.5
MR-ctrl_MN_rep2	10716514	10293398	96.0
MR-reg_MN_rep1	12144392	11646416	95.8
MR-reg_MN_rep2	8268676	7992674	96.6
LR-ctrl_MN_rep1	11086670	10755750	97.0
LR-ctrl_MN_rep2	11732148	11411698	97.2
LR-reg_MN_rep1	8603818	8192000	95.2
LR-reg_MN_rep2	10699724	10326310	96.5

E

	Early	Mid	Late
More accesible	9319	4732	3137
Less accessible	56	620	709

Statistics and replicate analysis of ATAC-seq. ATAC-seq was performed from two independent biological replicates from each time point and condition. (A) Scatter plots showing high correlation of peak heights between replicates (Pearson and Spearman correlation coefficients higher than 0.85, denoted by p_r and s_r, respectively). (B) Line plot showing read density per fragment length. Fragments belonging to nucleosome-free region (NF) fall in 0 to 100bp, meanwhile mononucleosome fraction (MN) fall in 180 to 247bp. (C) NF mapping statistics. (D) MN mapping statistics. (E) Number of differentially accessible regions at all time points.

Supplemental Figure S5

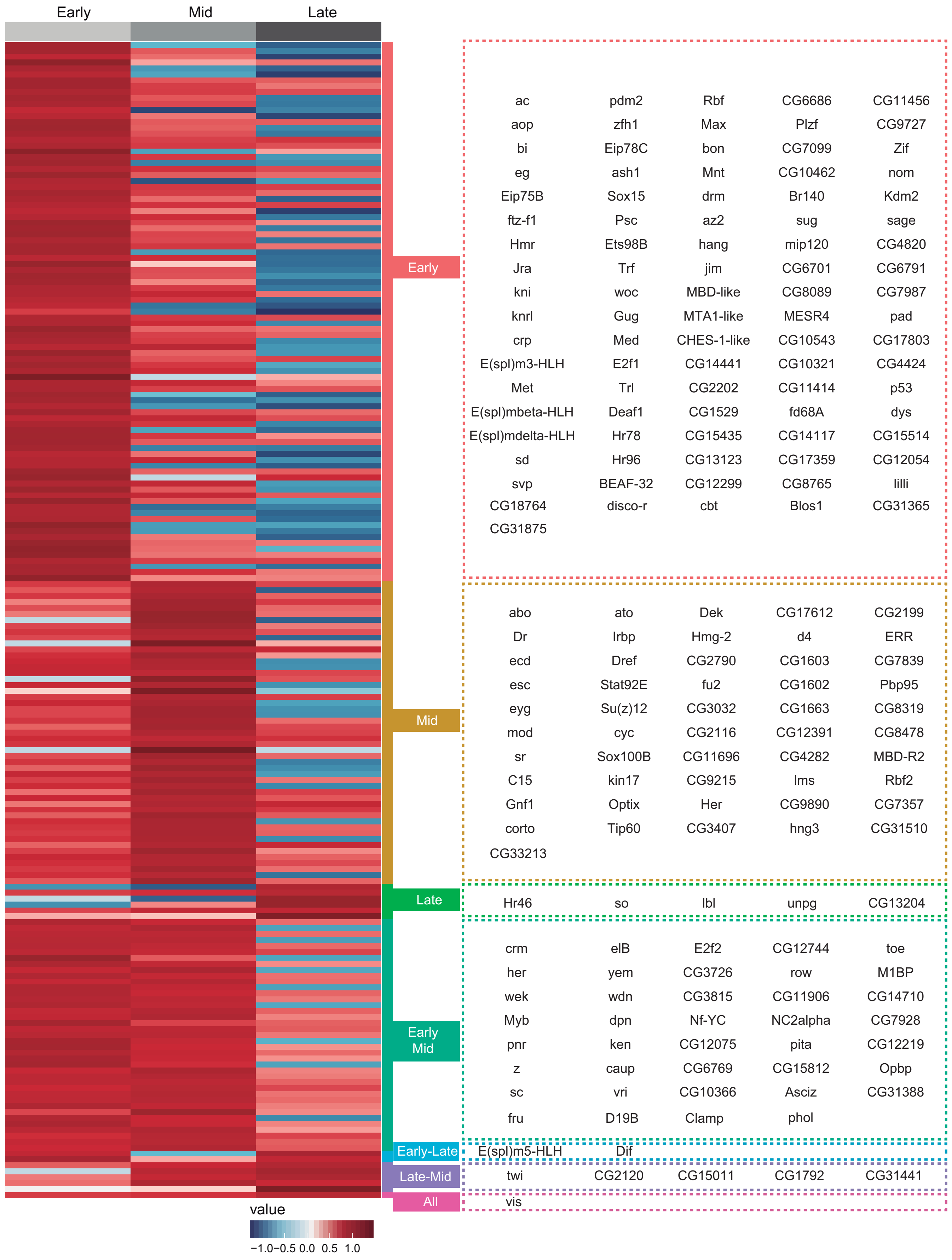

Expression profiles of upregulated transcription factors. Heatmap showing the expression fold change of genes encoding transcription factors upregulated in at least one time point throughout the recovery process. Gene names are shown.

Supplemental Figure S6

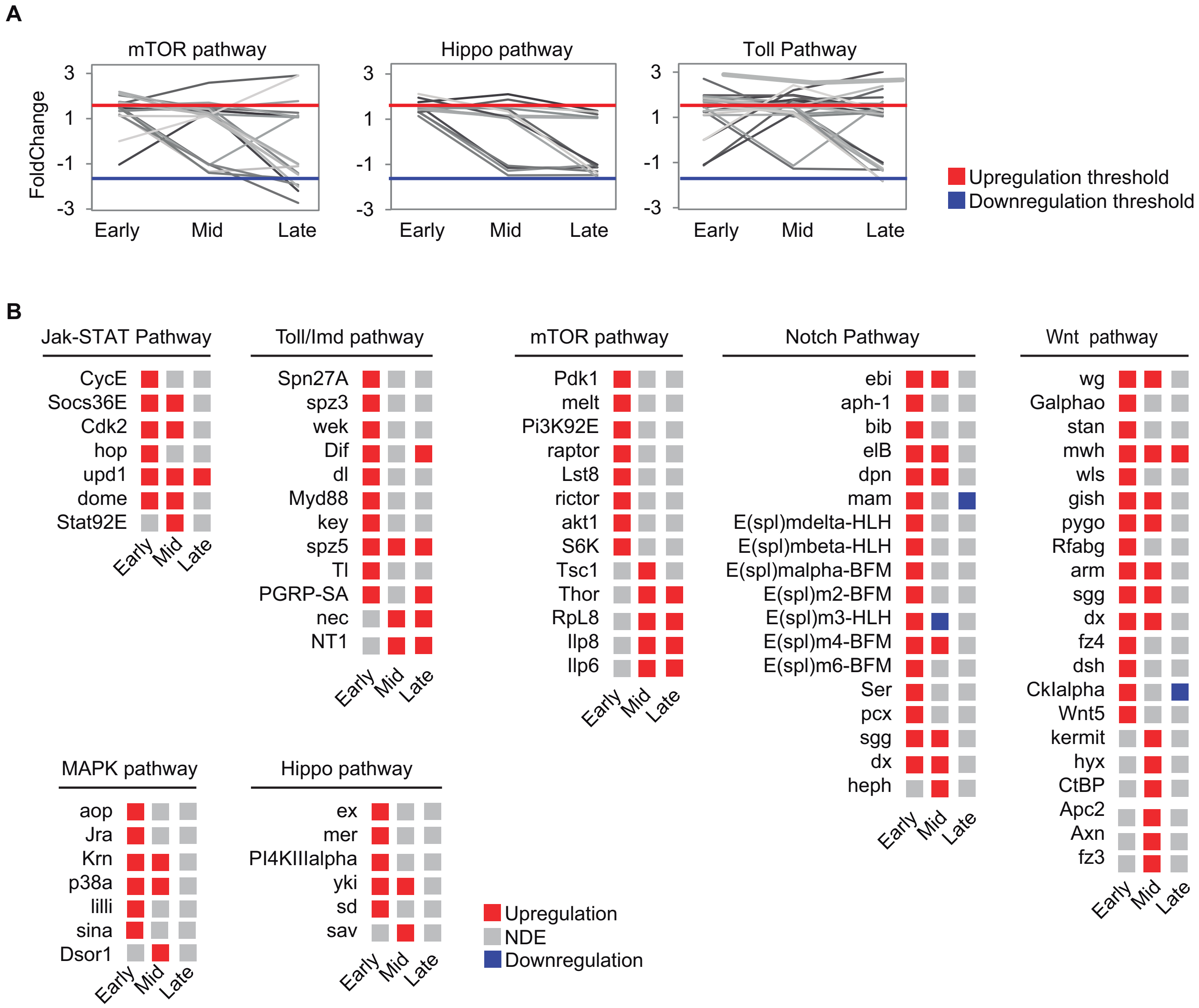

Pathway enrichment in upregulated genes. (A) Line plots showing expression changes over time of genes that belong to signaling pathways significantly enriched in regeneration. Expression is shown as fold change between control and regeneration at each time point. Each gene is plotted as a single line. (B) Expression profile of the upregulated members of the enriched pathways at different time points.

Supplemental Figure S7

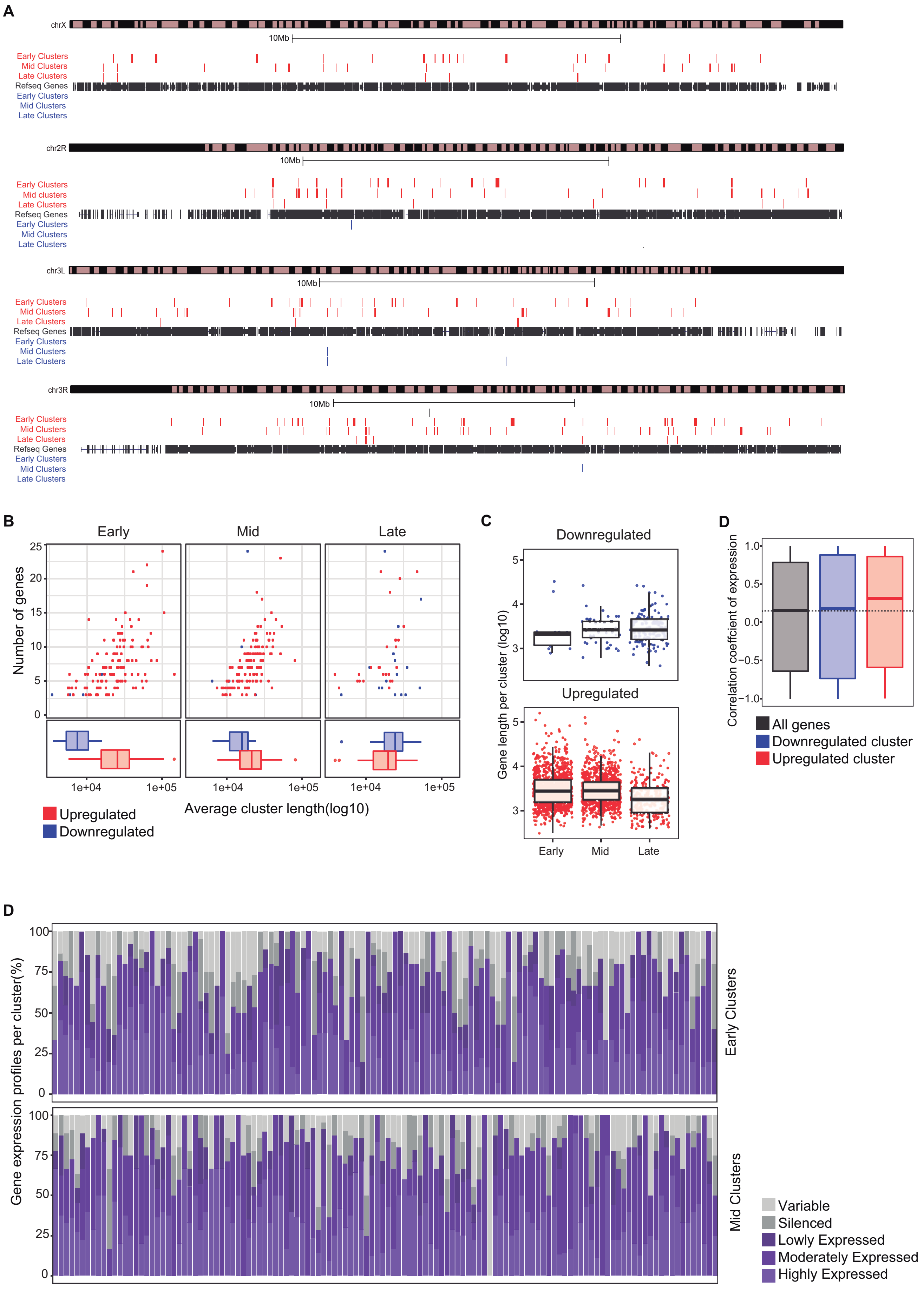

Features of genomic clusters. (A) Genomic map of clusters of differentially expressed genes on all chromosomes except 2L. Each red or blue box represents one single cluster. The size of each box denotes the length of each cluster. (B) Scatter plot showing the number of protein coding genes and the length of the cluster. Each dot represents a cluster (top). Box plot showing the average cluster length (bottom). (C) Box plot showing the average gene length per cluster. Each dot represents a cluster (Top). (D) Box plot showing the average Pearson correlation coefficient of gene expression through time. (D) Bar plot showing in percentage the average gene expression profile through time per cluster. Each bar represents one individual cluster.

Supplemental Figure S8

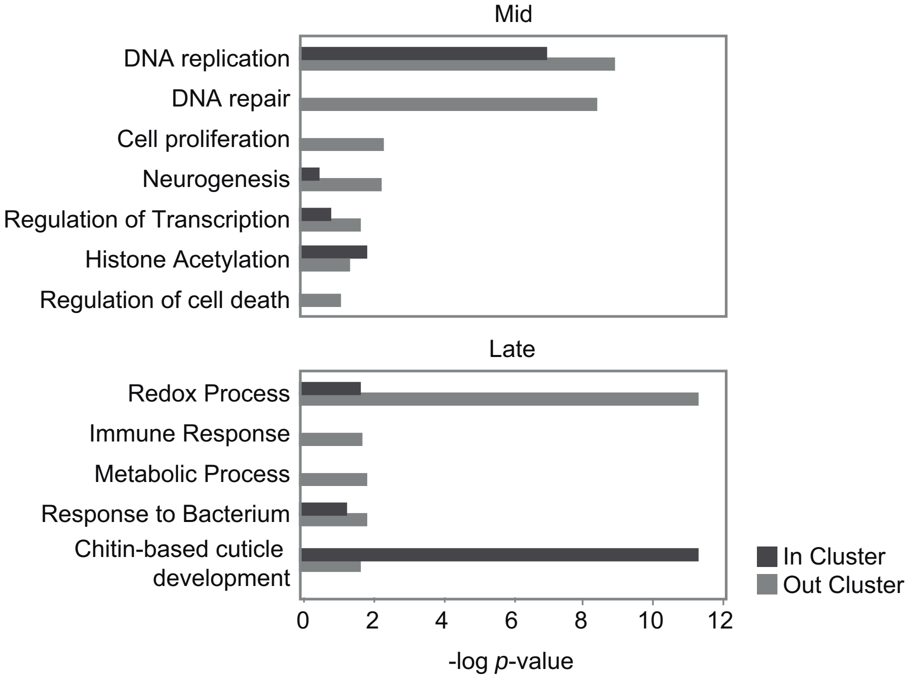

Gene Ontology of differentially expressed genes. GO term enrichment for the set of upregulated genes located inside or outside the clusters at mid and late time points. All the categories plotted are significant in at least one group of genes (the absence of a bar denotes no enrichment in that group).

Supplemental Figure S9

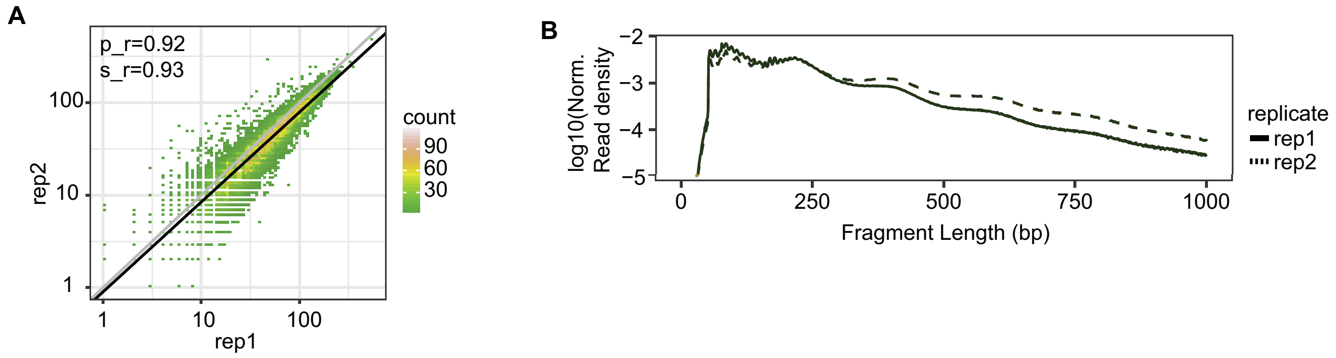

C

labExpId	total	uniq	prop_uniq
L3_NF_rep1	11888930	11610672	97.6
L3_NF_rep2	10593812	10224844	96.5
L3_MN_rep1	9610576	9330692	97.0
L3_MN_rep2	1243144	11886844	95.6

Statistics and replicate analysis of third instar larval ATAC-seq. (A) Scatter plot showing high correlation of peak height between replicates in L3 ATAC-seq (Pearson and Spearman correlation coefficients higher than 0.9, denoted by p_r and s_r, respectively). (B) Line plot showing read density per fragment length. Fragments belonging to NF will fall in 0 to 100bp meanwhile MN fraction will fall in 180 to 247bp. (C) NF and MN mapping statistics for L3.

Supplemental Figure S10

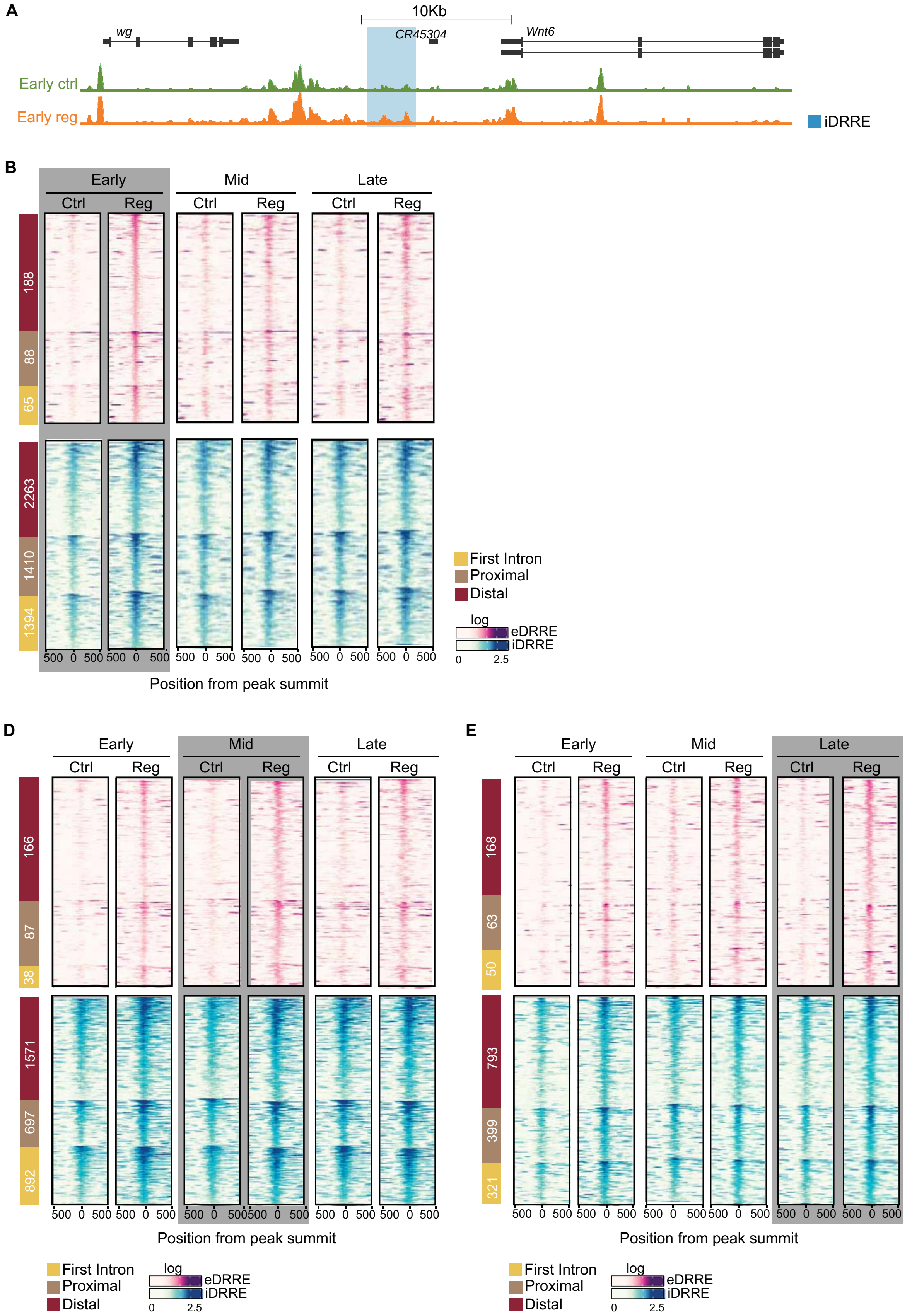

Accessible chromatin landscape after cell death induction. (A) Genome browser screenshot highlighting the BRV18-B region of the damage-activated WNT enhancer (Harris et al. 2016). This enhancer is now classified as a iDRRE. (B) Heatmaps showing NF regions around ±500bp of the peak summit of early DRREs displayed across time. (C) Heatmaps showing NF regions around ±500bp of the peak summit of mid DRREs displayed across time. (D) Heatmaps showing NF regions around ±500bp of the peak summit of late DRREs displayed across time. Sites are ordered by genomic distribution (shown in the left) and by peak height based on the ATAC-seq regeneration sample highlighted in gray.

Supplemental Figure S11

A

labExpId	total	uniq	prop_uniq
H3K4me1-Ctrl	13553454	2420784	93.23
H3K4me1-Reg	12826631	3976668	92.87
H3K27ac-Ctrl	10678716	6380199	97.26
H3K27ac-Reg	10073238	7396532	96.92
RNApol-Ctrl	20365397	2197057	92.7
RNApol-Reg	12992737	2094676	94.12

B

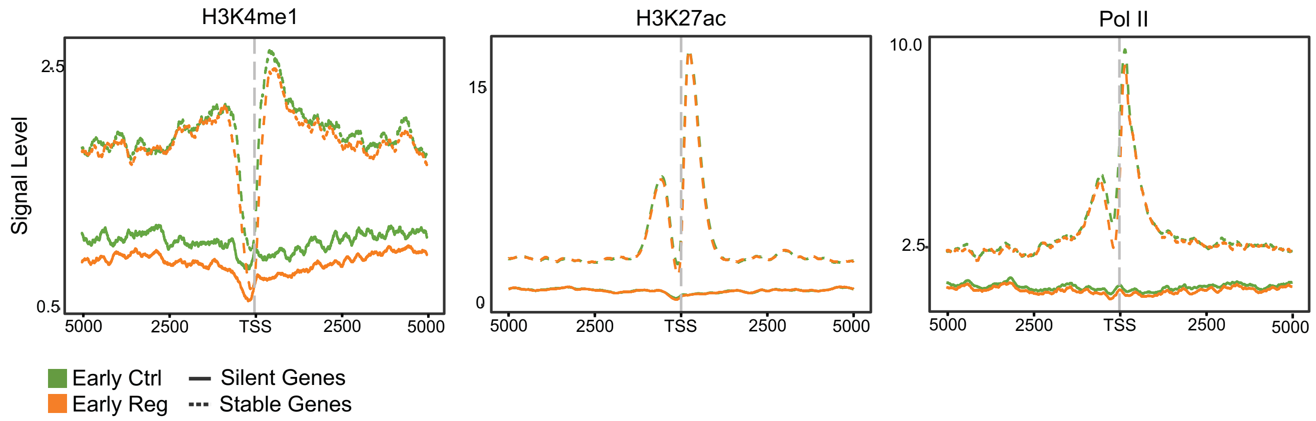

Statistics and analysis of ChIP-seq. (A) ChIP-seq mapping statistics for early control and regeneration. (B) Quality check for ChIP-seq based on the signal level at the TSS of modEncode stable and silent genes. Silent genes should have flat signal whereas stable genes should have clear signal (see Materials and Methods). Average profile of H3K4me1, H3K27ac and Pol II around ±5000 bp of the TSS of L3 wing disc modENCODE silent and active genes.

Supplemental Figure S12

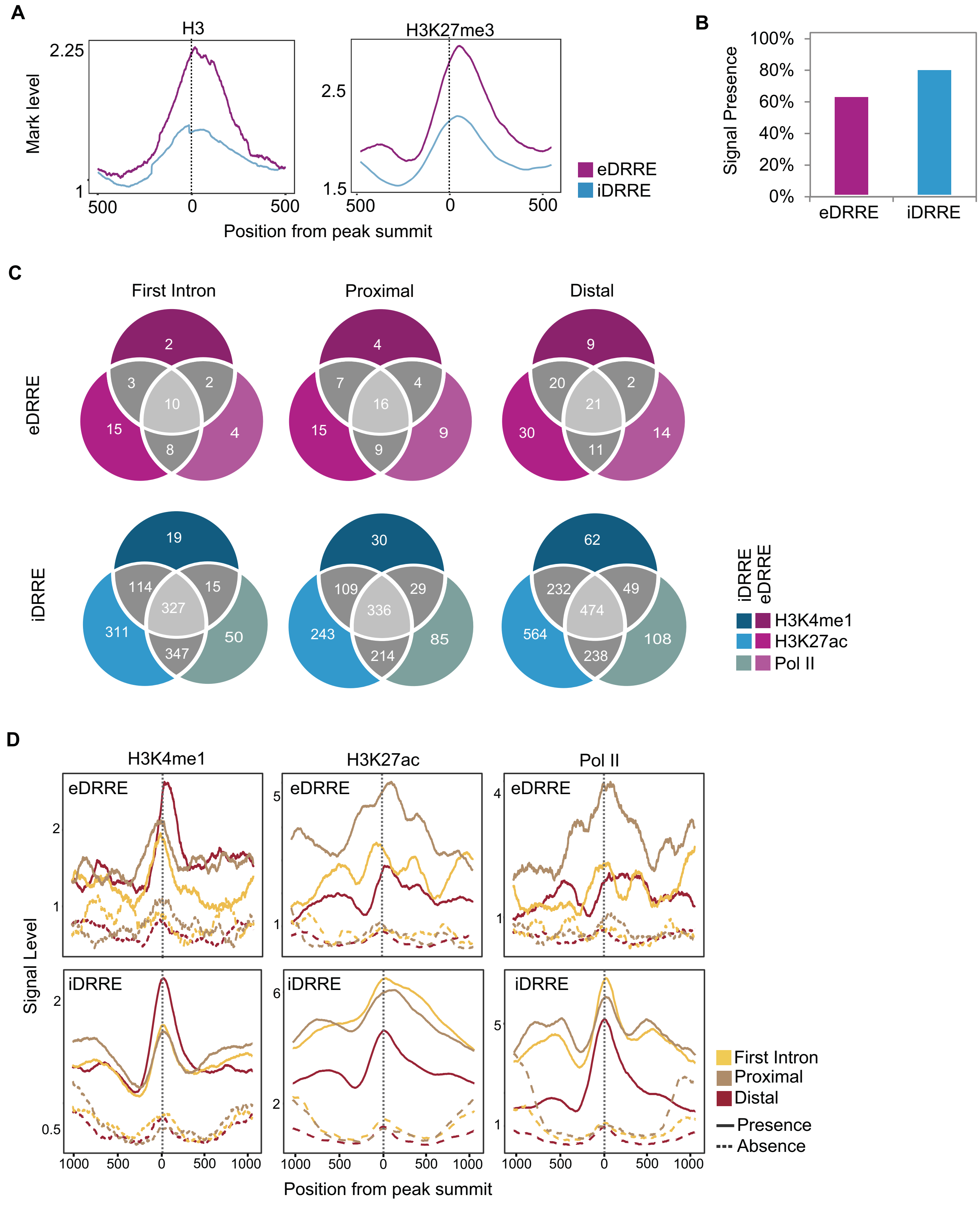

Chromatin features of DRREs. (A) Average profile of H3 and H3K27me3 around ±500bp of the peak summit of DRREs at L3 wing discs. (B) Bar plot showing the percentage of presence or absence of at least one ChIP-seq signal at DRREs. (C) Venn diagrams showing the intersection of ChIP signal in DRREs per genomic distribution. (D) Average profile of ChIP-seq signal around ±1000bp of the peak summit of DRREs in regeneration. A solid line denotes DRREs with presence of at least one ChIP-seq signal and a dashed line denotes absence of any ChIP-seq signal.

Supplemental Figure S13

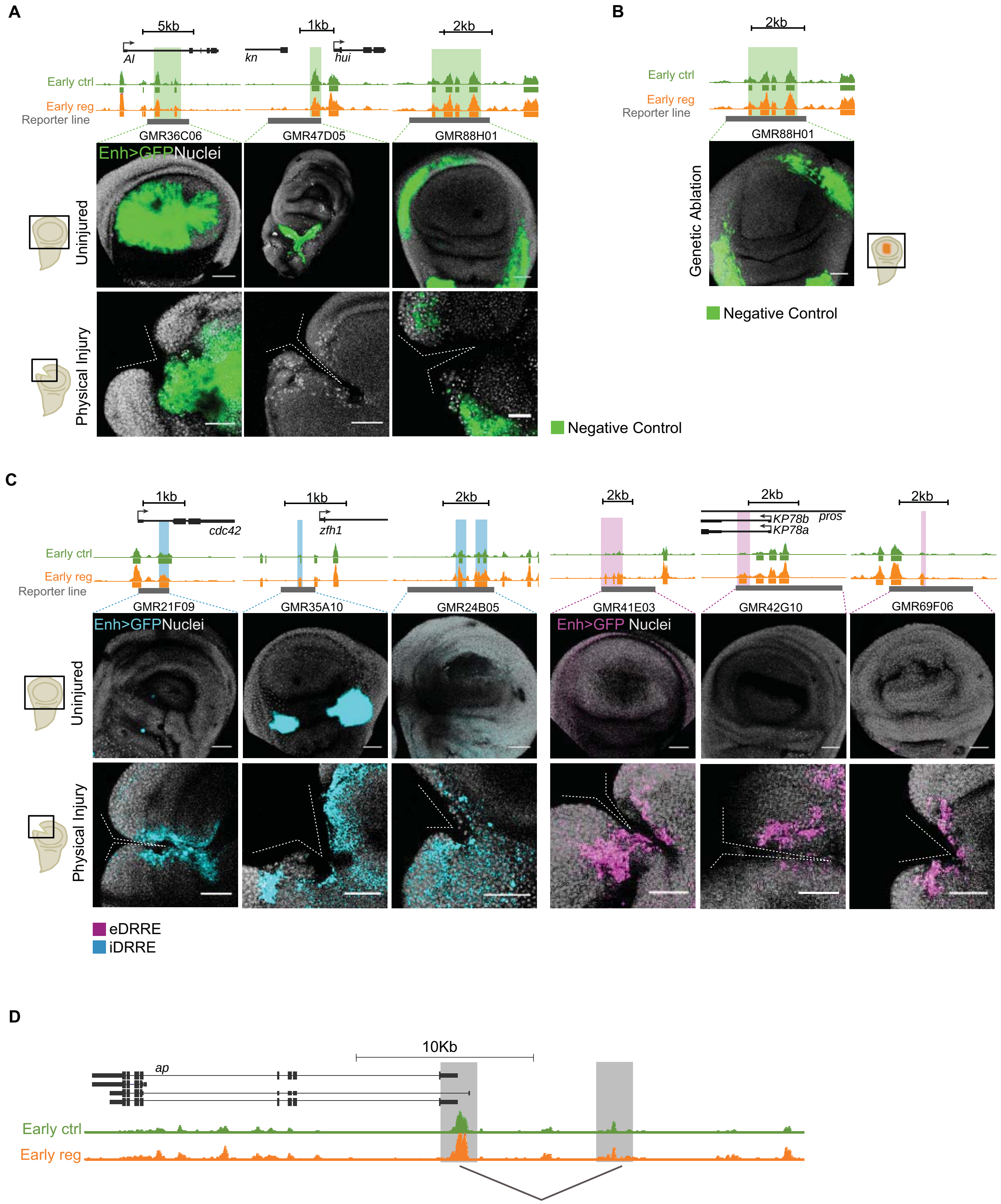

Validation of the activity of DRREs after damage. (A, B) Reporter lines containing regions not showing differential accessibility after damage and used as negative controls. (C) Validation of DRREs after physical damage using reporter lines. Genome Browser screenshot showing the ATAC-seq profile (control and regeneration) at the early regeneration of validated enhancers (highlighted in green if negative control; blue if iDRRE; purple if eDRRE), and the region covered by the reporter line in gray (top). Confocal images of wing discs showing enhancer activity as GFP intensity (bottom). The injury domain is shown in a schematic drawing in the right. (D) Genome Browser screenshot depicting the interaction of a known enhancer on the *Apterous* (*ap*) gene (Bieli et al. 2015) used as a control on 3C experiments.

Supplemental Figure S14

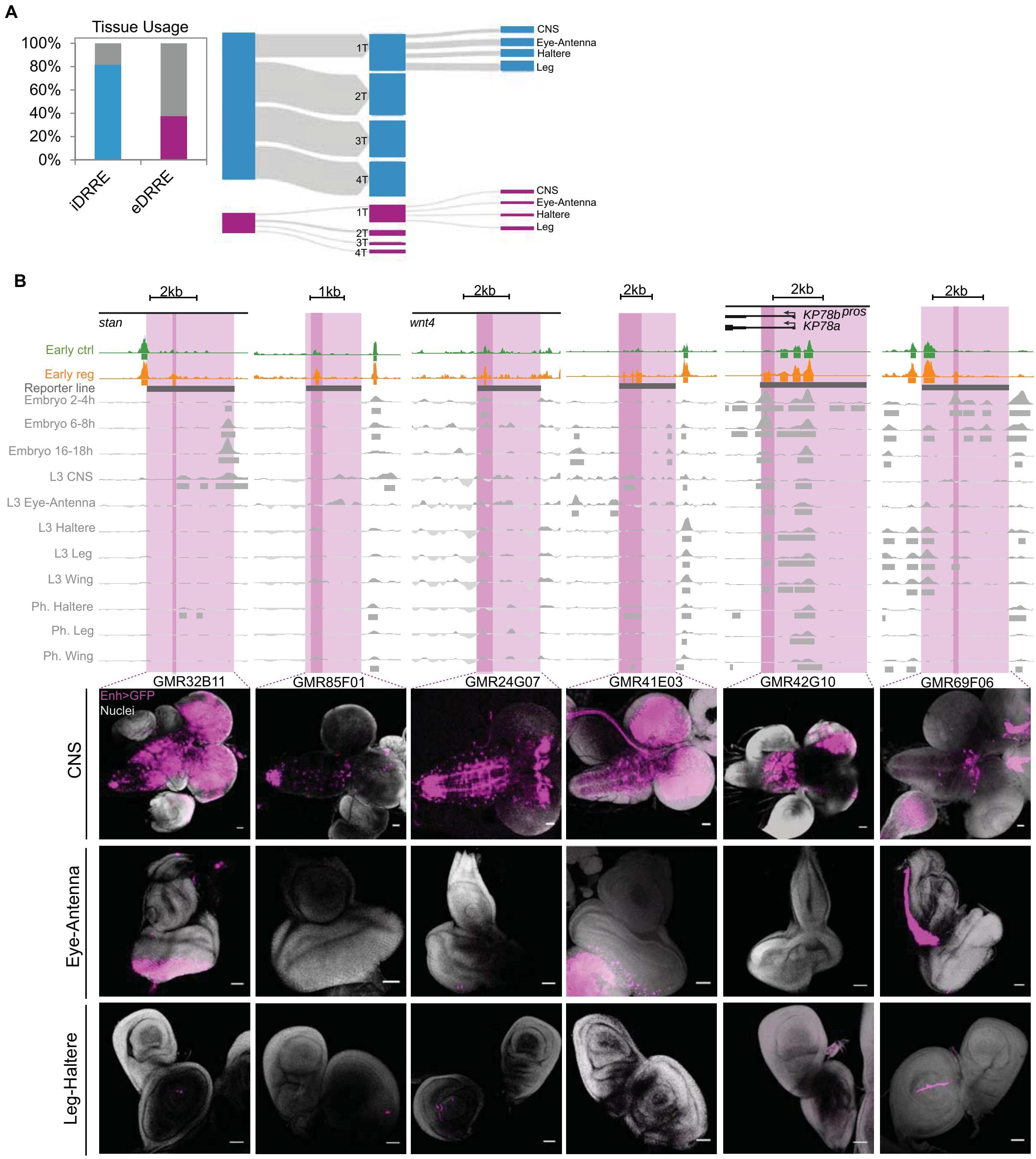

Tissue usage of DRREs. (A) Flux plot of enhancers activated after damage showing their usage in other tissues at L3 stage. DRRE peaks are classified in base of their presence in tissues (Central Nervous system – CNS, eye-antenna disc, haltere disc and leg disc) and in the amount of occasions each enhancer is employed (up to 4 tissues - T). (B) Tissue usage of reporter lines. Genome Browser screenshot showing the ATAC-seq profile (control and regeneration) at early regeneration of validated eDRREs (highlighted in dark purple), the FAIRE profiles (in gray) and the region covered by the reporter line (highlighted in light purple) (top). Confocal images of CNS, eye-antenna discs, leg discs and haltere discs at L3 stage showing enhancer activity as GFP intensity (bottom).

Supplemental Figure S15

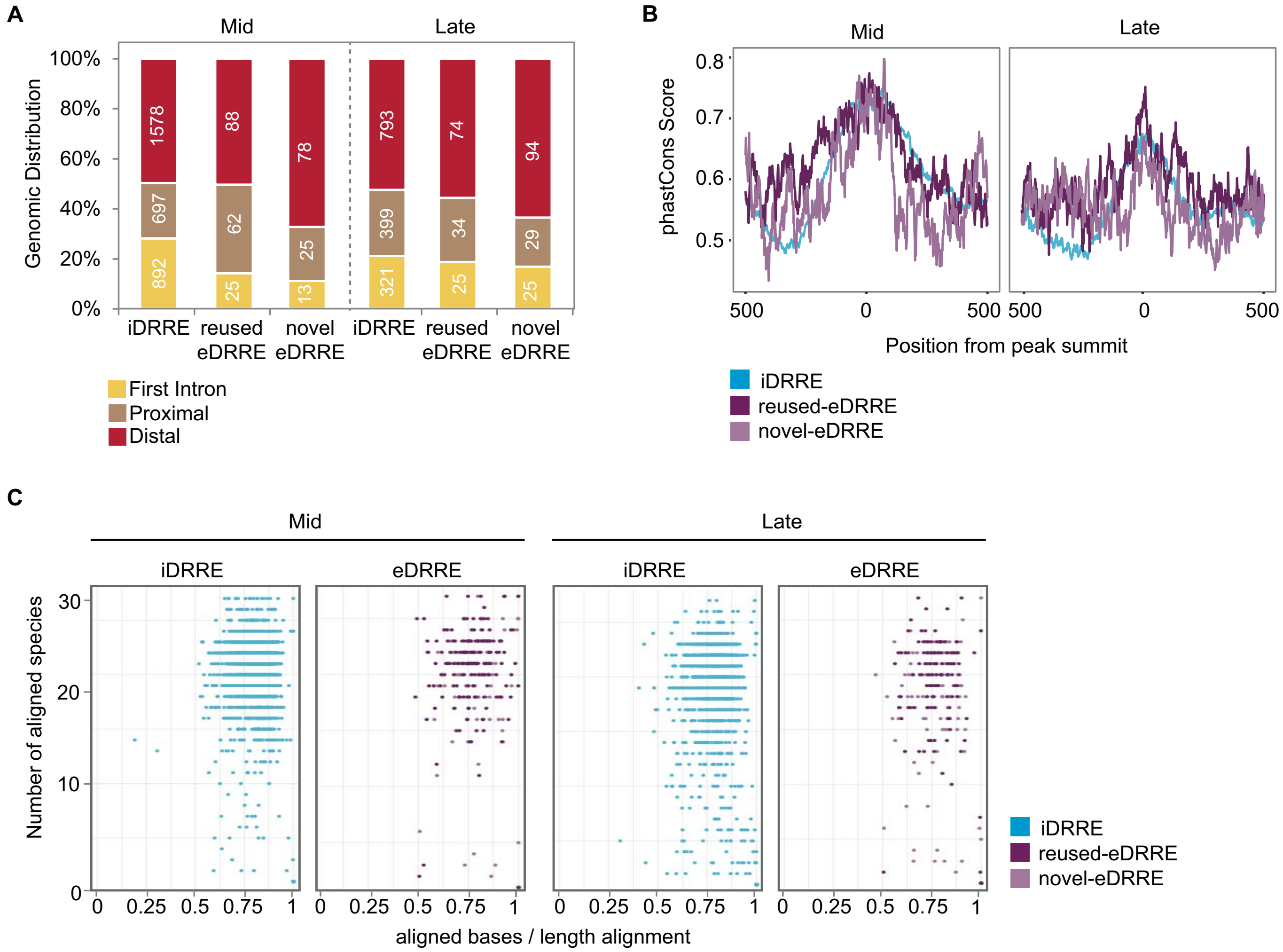

Reusage and conservation of DRREs at mid and late time points. (A) Genomic distribution of DRREs: first intron, proximal and distal. (B) Average distribution of PhastCons scores derived from 27 insect species in the DRRE sequences (defined as 500 bp upstream and downstream of the NF peak summit). (C) Conservation of DRREs across 27 insect species. Each dot corresponds to one independent enhancer. The Y-axis denotes the number of species that present the conserved enhancer. The X-axis represents the percentage of aligned bases per sequence length.

Supplemental Figure S16

A

STARR-seq activation in (n)spcies		1 Species	2 Species	3 Species	4 Species	NA
Early	iDRRE	623	494	563	728	2659
	reused-eDRRE	27	20	10	0	141
	novel-eDRRE	17	8	6	1	111

Total STARR-seq activation in each species		*D. yak*	*D. ana*	*D. pse*	*D. will*	NA
Early	iDRRE	1544	1564	1641	1463	2659
	reused-eDRRE	27	21	21	28	141
	novel-eDRRE	15	12	16	12	111

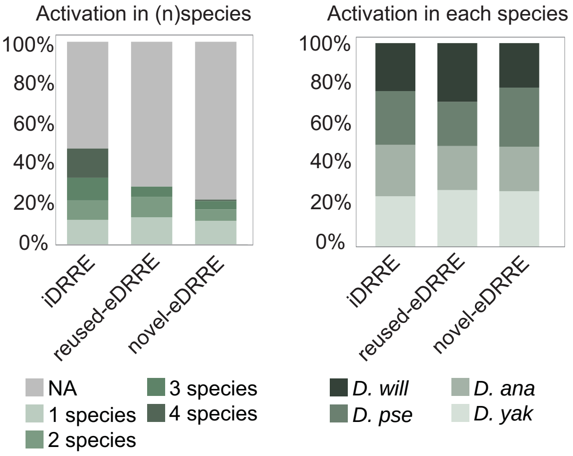

B

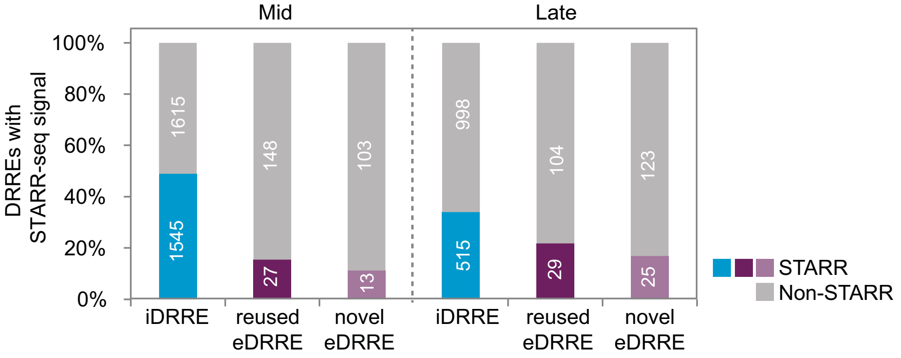

C

STARR-seq activation in (n)species	DRRE type	1 Species	2 Species	3 Species	4 Species	NA
Mid	iDRRE	407	314	281	543	1615
	reused-eDRRE	16	8	2	1	148
	novel-eDRRE	5	7	1	0	103
Late	iDRRE	192	115	102	106	998
	reused-eDRRE	14	9	6	0	104
	novel-eDRRE	11	8	5	1	123

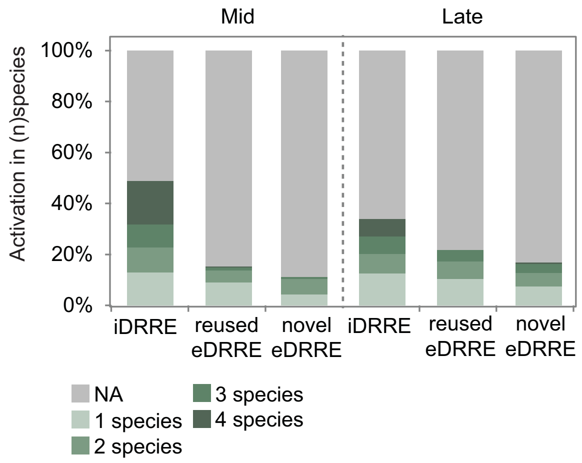

Conservation of DRREs. (A) Tables showing the number of species containing the same active enhancer (top) and the number of active enhancers present in each species (bottom) at the early time point. Bar plots showing the same numbers in percentage (right). (B) Percentage of conserved DRREs that are active at mid and late time points, according to the STARR-seq technique. (C) Tables showing the number of species containing the same active enhancer at the mid and late time points (left). Bar plots showing the same numbers in percentage (right).

Supplemental Figure S17

A

	Early			Mid			Late		
	up	NDE	down	up	NDE	down	up	NDE	down
Mapped in both species	1269	5880	54	1154	5972	77	227	6625	351
Only mapped in zebrafish	19	133	2	23	128	3	5	141	8
Only mapped in mouse	18	83	0	14	86	1	5	92	4
non-mapped	691	5667	104	440	5909	113	380	5891	191
total	1997	11763	160	1631	12095	194	617	12749	554
% mapped	65.3980971	51.8235144	35	73.022685	51.1451013	41.7525773	38.411669	53.7924543	65.523466

	Chi-Value		*p*-value	
	up	down	up	down
Early	126.56	17.889	0	2.342E-05
Mid	276.5	6.74	0	0.0094
Late	55.9	29.44	0	6E-08

B

Orthologs genes ratios			
		Zebrafish	Mouse
1	Mapped fly genes	7357	7304
2	All fly orthologs upregulated in	2476	2347
	Ratio 2/1	33.66%	32.13%
3	Upregulated fly genes mapped	1288	1287
4	Upregulated fly genes mapped to an upregulated gene in	419	431
	Ratio 3/4	32.53%	33.49%

C

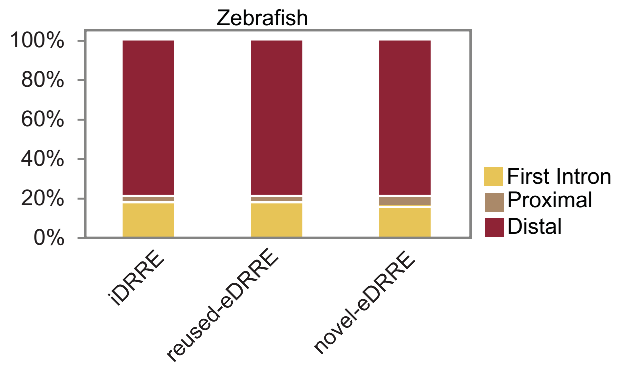

Homology of genes implicated in fly regeneration. (A) Table showing the number and percentage of DE fly genes that map to an ortholog in zebrafish, mouse or in both. Also it is shown the Chi-value and the *p*-value of the comparison between non-differentially expressed (NDE) and upregulated or downregulated genes in each time point. (B) Table showing ratios based on mapping statistics of shared regenerative genes. (C) Bar plot showing genomic distribution of the three types of DRREs identified in zebrafish.

SUPPLEMENTAL FIGURES REFERENCES

Bieli D, Kanca O, Requena D, Hamaratoglu F, Gohl D, Schedl P, Affolter M, Slattery M, Müller M, Estella C. 2015. Establishment of a Developmental Compartment Requires Interactions between Three Synergistic Cis-regulatory Modules. *PLOS Genet* **11**: e1005376.

Harris RE, Setiawan L, Saul J, Hariharan IK. 2016. Localized epigenetic silencing of a damage-activated WNT enhancer limits regeneration in mature Drosophila imaginal discs. *Elife* **5**.
