# Supplementary material for: Damage-responsive elements in Drosophila regeneration
Source: Genome Res. 2018 Dec;28(12):1852–66. doi: 10.1101/gr.233098.117 (PMC6280756; doi:10.1101/gr.233098.117)
Supplement: Supplemental Material [file supp_gr.233098.117_Supplemental_Methods.pdf]

SUPPLEMENTAL METHODS FOR

“Damage responsive elements in *Drosophila* regeneration”

Elena Vizcaya-Molina, Cecilia C. Klein, Florenci Serras, Rakesh Mishra, Roderic Guigó and

Montserrat Corominas

TABLE OF CONTENTS

Stock List

Experiment Genotypes

Primer List

Supplemental Methods References

Supplemental Methods

Reporter Lines				
DRRE Coordinates	DRRE type	Reporter Line	Resource	Code
chrX:19697549-19697888	iDRRE	GMR21F09	Bloomington Stock Center	46164
chr3R:30765485-30765822	iDRRE	GMR35A10	Bloomington Stock Center	49897
chr2L:7286945-7287225	iDRRE	GMR17D09	Bloomington Stock Center	48766
chr3L:6216670-6217317	iDRRE	GMR25D02	Bloomington Stock Center	45848
chr3R:10437127-10437322	iDRRE	VT39456	Viena Drosophila RNAi Center	VT39456
chrX:16254669-16254835	iDRRE	GMR26G03	Bloomington Stock Center	49169
chr2R:10680648-10680812	eDRRE	GMR32B11	Bloomington Stock Center	47539
chr2L:11472301-11472450	eDRRE	GMR85E02	Bloomington Stock Center	46801
chr2L:7266527-7266631	eDRRE	GMR24G07	Bloomington Stock Center	49095
chr3R:11351859-11352162	eDRRE	GMR42G10	Bloomington Stock Center	50168
chr2R:8640900-8641040	eDRRE	GMR69F06	Bloomington Stock Center	39497
chr2R:5741496-5741591	eDRRE	GMR41E03	Bloomington Stock Center	50126
chr3R:13938246-13938465	Neg Ctrl	GMR36C06	Bloomington Stock Center	49931
chr2R:14807712-14808131	Neg Ctrl	GMR47D05	Bloomington Stock Center	47605
chr3R:13939143-13939310	Neg Ctrl	GMR88H01	Bloomington Stock Center	40529

Drosophila Strains		
Line	Resource	Code
w^{1118}	Bloomington Stock Center	5905
UAS-rpr	Wing et al. 1999	NA
LexO-rpr	Santabárbara-Ruiz et al. 2015	NA
salm-Gal4	Bergantiños et al. 2010	NA
$sal^{E/PV}$-LHG	Santabárbara-Ruiz et al. 2015	NA
tub-$Gal80^{TS}$	McGuire et al. 2003	NA
ci-Gal4	Martin and Morata 2006	NA
UAS-mCD8GFP	Bloomington Stock Center	32186
RNAi-Dif	Viena Drosophila RNAi Center	V100537
RNAi-Lilli	Viena Drosophila RNAi Center	V106142-KK
RNAi-Stat92-E	Viena Drosophila RNAi Center	V4386G-GD

Stock List. List of all the stocks acquired from VDRC and Bloomington.

Supplemental Methods

Experiment Genotypes		
Fig 1A,B	Control	w;salm-Gal4;tub-Gal80^{TS}
	Regenaration	wUAS-rpr;salm-Gal4;tub-Gal80^{TS}
Fig 4A	Control - iDRRE	UAS-mCD8GFP/+;GMR25D02-Gal4 /+
	Control - iDRRE	UAS-mCD8GFP/+;VT39456-Gal4/+
	Control - iDRRE	UAS-mCD8GFP/+; GMR26G03-Gal4/+
	Control - eDRRE	UAS-mCD8GFP/+;GMR32B11-Gal4/+
	Control - eDRRE	UAS-mCD8GFP/+;GMR85E02-Gal4/+
	Control - eDRRE	UAS-mCD8GFP/+;GMR24G07-Gal4/+
	Injury - iDRRE	UAS-mCD8GFP/+;GMR25D02-Gal4 /+
	Injury - iDRRE	UAS-mCD8GFP/+;VT39456-Gal4/+
	Injury - iDRRE	UAS-mCD8GFP/+; GMR26G03-Gal4/+
	Injury - eDRRE	UAS-mCD8GFP/+;GMR32B11-Gal4/+
	Injury - eDRRE	UAS-mCD8GFP/+;GMR85E02-Gal4/+
	Injury - eDRRE	UAS-mCD8GFP/+;GMR24G07-Gal4/+
	Genetic Ablation - iDRRE	UAS-mCD8GFP/lexO-rpr;GMR25D02-Gal4/sal$^{E/PV}$-LHG:tub-Gal80^{TS}
	Genetic Ablation - iDRRE	UAS-mCD8GFP/lexO-rpr;VT39456-Gal4 /sal$^{E/PV}$-LHG:tub-Gal80^{TS}
	Genetic Ablation - iDRRE	UAS-mCD8GFP/lexO-rpr;GMR26G03-Gal4 /sal$^{E/PV}$-LHG:tub-Gal80^{TS}
	Genetic Ablation - eDRRE	UAS-mCD8GFP/lexO-rpr;GMR32B11-Gal4/sal$^{E/PV}$-LHG:tub-Gal80^{TS}
	Genetic Ablation - eDRRE	UAS-mCD8GFP/lexO-rpr;GMR85E02-Gal4/sal$^{E/PV}$-LHG:tub-Gal80^{TS}
	Genetic Ablation - eDRRE	UAS-mCD8GFP/lexO-rpr;GMR24G07-Gal4/sal$^{E/PV}$-LHG:tub-Gal80^{TS}
Fig 4B	Control	w;salm-Gal4;tub-Gal80^{TS}
	Regenaration	wUAS-rpr;salm-Gal4;tub-Gal80^{TS}
Fig 6D	Cell Death ON, RNAi OFF	ci-Gal4/+;lexO-rpr/sal$^{E/PV}$-LHG:tub-Gal80^{TS}
	Cell Death OFF, RNAi ON	ci-Gal4/RNAi-Dif;sal$^{E/PV}$-LHG:tub-Gal80^{TS}/+
	CellDeath OFF, RNAi ON	ci-Gal4/RNAi-Stat92-E;sal$^{E/PV}$-LHG:tub-Gal80^{TS}/+
	Cell Death OFF, RNAi ON	ci-Gal4/RNAi-Lilli;sal$^{E/PV}$-LHG:tub-Gal80^{TS}/+
	Cell Death ON, RNAi ON	ci-Gal4/RNAi-Dif;lexO-rpr/sal$^{E/PV}$-LHG:tub-Gal80^{TS}
	Cell Death ON, RNAi ON	ci-Gal4/RNAi-Stat92-E;lexO-rpr/sal$^{E/PV}$-LHG:tub-Gal80^{TS}
	Cell Death ON, RNAi ON	ci-Gal4/RNAi-Lilli;lexO-rpr/sal$^{E/PV}$-LHG:tub-Gal80^{TS}
Suppl Fig 9A	Control - NegCtrl	UAS-mCD8GFP/+;GMR36C06-Gal4/+
	Control - NegCtrl	UAS-mCD8GFP/+;GMR47D05-Gal4/+
	Control - NegCtrl	UAS-mCD8GFP/+;GMR88H01-Gal4/+
	Injury - NegCtrl	UAS-mCD8GFP/+;GMR36C06-Gal4/+
	Injury - NegCtrl	UAS-mCD8GFP/+;GMR47D05-Gal4/+
	Injury - NegCtrl	UAS-mCD8GFP/+;GMR88H01-Gal4 /+
Suppl Fig 9B	Control - iDRRE	UAS-mCD8GFP/+;GMR21F09-Gal4/+
	Control - iDRRE	UAS-mCD8GFP/+;GMR35A10-Gal4/+
	Control - iDRRE	UAS-mCD8GFP/+;GMR17D09-Gal4 /+
	Control - eDRRE	UAS-mCD8GFP/+;GMR42G10-Gal4/+
	Control - eDRRE	UAS-mCD8GFP/+;GMR69F06-Gal4/+
	Control - eDRRE	UAS-mCD8GFP/+;GMR41E03-Gal4/+
	Injury - iDRRE	UAS-mCD8GFP/+;GMR21F09-Gal4 /+
	Injury - iDRRE	UAS-mCD8GFP/+;GMR35A10-Gal4/+
	Injury - iDRRE	UAS-mCD8GFP/+;GMR17D09-Gal4/+
	Injury - eDRRE	UAS-mCD8GFP/+;GMR42G10-Gal4/+
	Injury - eDRRE	UAS-mCD8GFP/+;GMR69F06-Gal4/+
	Injury - eDRRE	UAS-mCD8GFP/+;GMR41E03-Gal4/+
Suppl Fig 10B	reused-eDRRE	UAS-mCD8GFP/+;GMR32B11-Gal4/+
	reused-eDRRE	UAS-mCD8GFP/+;GMR85E02-Gal4/+
	reused-eDRRE	UAS-mCD8GFP/+;GMR24G07-Gal4/+
	reused-eDRRE	UAS-mCD8GFP/+;GMR42G10-Gal4/+
	reused-eDRRE	UAS-mCD8GFP/+;GMR69F06-Gal4/+
	reused-eDRRE	UAS-mCD8GFP/+;GMR41E03-Gal4/+

Experiment genotypes. List of all the genotypes used throughout the manuscript.

Supplemental Methods

3C-qPCR		
Interaction	Forward	Reverse
Distal: 2 with 1	TATACTCTGGCCTTCTGCAT	TGTGTCACGCATACGCAATAT
Distal: 2 with 5	ACTCTGCCTCACCGCATTA	GGGATACGTACAAGAATACCATAC
Distal: 2 with 4	ATACTGCGACACACAGTGC	CAAAATGAGTTGGCGGGACT
Distal: 2 with 3	ATACTGCGACACACAGTGC	TGAGACGGAGTGGCGTAAT
Distal: 3 with 4	ATACTGCGACACACAGTGC	CAGGAACAGCTACGGGATT
Distal: 4 with 5	TCCTTGTCGATGCCCTAAA	GTGAGACGGAGTGGCGTAA
Proximal: 2 with 1	GGCCGGAATGGAGGCACTT	ACGCCTCTGATCTCTGTACCG
Proximal: 3 with 1	GGTGTCGGTGAGAGAGTGTGATG	CTACGCCTCTGATCTCTGTACCG
Control: Apterous	AACATACTCTTCTCGCCCAATCC	ACGTTTCACTTCGAGTACGACGG

ChIP-qPCR		
Region	Forward	Reverse
WNT iDRRE	ACAGAAACCTCGCATTGCACTTT	TGCGAATTTGGAGTGATGGGTG
Proximal eDRRE	TGGTCAGTTGGGCTAGTGGA	TAGACGAGGTTGGCTATAATCT
Distal eDRRE	TTTGACATTGGTTCGGGCCT	CGGGCCTGCAACAGGTAATG

Primer List. List of all the primers used for 3C-qPCR and ChIP-seq experiments.

SUPPLEMENTAL METHODS REFERENCES

Bergantiños C, Corominas M, Serras F. 2010. Cell death-induced regeneration in wing imaginal discs requires JNK signalling. *Development* **137**: 1169–79.

Martín FA, Morata G. 2006. Compartments and the control of growth in the Drosophila wing imaginal disc. *Development* **133**: 4421–6.

McGuire SE, Le PT, Osborn AJ, Matsumoto K, Davis RL. 2003. Spatiotemporal rescue of memory dysfunction in Drosophila. *Science* **302**: 1765–8.

Santabárbara-Ruiz P, López-Santillán M, Martínez-Rodríguez I, Binagui-Casas A, Pérez L, Milán M, Corominas M, Serras F. 2015. ROS-Induced JNK and p38 Signaling Is Required for Unpaired Cytokine Activation during Drosophila Regeneration. *PLoS Genet*.

Wing JP, Zhou L, Schwartz LM, Nambu JR. 1999. Distinct cell killing properties of the Drosophila reaper, head involution defective, and grim genes. *Cell Death Differ* **6**: 212–213.
