# Supplementary material for: Damage-responsive elements in Drosophila regeneration
Source: Genome Res. 2018 Dec;28(12):1852–66. doi: 10.1101/gr.233098.117 (PMC6280756; doi:10.1101/gr.233098.117)
Supplement: Supplemental Material [file supp_gr.233098.117_Supplemental_Table_Legends.pdf]

SUPPLEMENTAL TABLES FOR

“Damage responsive elements in *Drosophila* regeneration”

Elena Vizcaya-Molina, Cecilia C. Klein, Florenci Serras, Rakesh Mishra, Roderic Guigó and Montserrat Corominas

TABLE OF CONTENTS

Supplemental Table S1 - RNA-seq analysis and classification

Supplemental Table S2 - List of genomic clusters and cluster hotspots

Supplemental Table S3 - ATAC-seq analysis and classification

Supplemental Table S4 - ATAC-seq comparative analysis

Supplemental Table S5 - Motif discovery in DRREs

Supplemental Table S6 - List of conserved TFs upregulated in regeneration

Supplemental Table S1

RNA-seq analysis and classification. Each gene was identified by its FlyBase ID, gene symbol and gene type according to FlyBase annotation r6.05 (columns 1-3). Pairwise classification performed for gene expression fold change between regeneration and control for early, mid and late time points (columns 4-6). Genes inside genomic clusters were classified based on the cluster category (column 7). Genes were assigned to the following GO biological processes when applicable: GO:0007259 JAK-STAT cascade, GO:0008063 Toll signaling pathway, GO:0007219 Notch signaling pathway, GO:0000165 MAPK cascade, GO:0016055 Wnt signaling pathway, GO:0008286 insulin receptor signaling pathway, GO:0035329 hippo signaling, GO:0031929 TOR signaling (column 8).

Supplemental Table S2

List of genomic clusters and cluster hotspots. Table showing the coordinates, the *p*-value and gene names of differentially expressed genes in each cluster.

Supplemental Table S3

ATAC-seq analysis and classification. Each nucleosome-free ATAC-seq peak was identified by an ID and genomic coordinates (columns 1-2). Peaks were classified by genomic distribution (core-promoter, first-intron, proximal and distal from TSS as described in Material and Methods) and genomic context (intergenic, exonic, intronic) in columns 3 and 4, respectively. Pairwise classification of peaks between regeneration and control for early, mid and late time points, where (i) eDRRE: peaks called exclusively in regeneration samples and concordant between replicates per time point and no peak called in control or L3; and (ii) iDRRE: peaks called in all samples, but higher in regeneration compared to control; see Material and Methods; columns 5-7). L3 concordance of peaks was used for classifying DRRE (column 8). Active enhancer features included H3K4me1, H3K27ac and PolII (columns 9-11), where the classification for each feature was as follows (i) *marked:* presence of a peak in regeneration samples in a window of 500bp up/downstream the ATAC-seq peak; (ii) *higher signal:* average signal is higher in regeneration compared to control sample in the same time point; and (iii) *not marked:* none of the previously described cases. Final active enhancer classification (column 12) was based on: (i) *presence:* at least one of the features was either classified as marked or higher signal; (ii) *absence:* all features were classified as *not marked*.

Supplemental Table S4

ATAC-seq comparative analysis. Each DRRE was identified by an ID and genomic coordinates (columns 1-2). Peaks were classified by genomic distribution (core-promoter, first-intron, proximal and distal from TSS as described in Material and Methods; column 3). Pairwise classification of peaks between regeneration and control for early, mid and late time points, where (i) eDRRE: peaks called exclusively in regeneration samples and concordant between replicates per time point and no peak called in control or L3; (i.1) reused-eDRRE: used in other tissues or across different developmental stages and (i.2) novel-eDRRE: not used in other tissues or across different developmental stages and (ii) iDRRE: peaks called in all samples, but higher in regeneration compared to control; see Material and Methods; columns 4-6). We assessed whether DRREs were involved in other developmental events using chromatin accessibility data (Nègre et al. 2011) from different tissues and stages of fly development (columns 7-8). DRRE conservation was studied using the phastCons measurement of evolutionary conservation and the dm6 27-way multiple alignment (23 *Drosophila* sequences, house fly, *Anopheles* mosquito, honey bee and red flour beetle) from the UCSC Genome Browser (Tyner et al. 2017) and STARR-seq data (Arnold et al. 2014) on genome-wide enhancer activity profiles for five *Drosophila* species (columns 9-12).

Supplemental Table S5

Motif discovery in DRREs. Tables showing all the TFs putatively binding to an enriched motif in DRREs at the different time points. For the motifs found at the early stage, TFs that are upregulated in regeneration are shown in bold and TFs that are unique to each DRRE and underlined.

Supplemental Table S6

List of conserved TFs upregulated in regeneration. (A) Table showing the list of mapped and upregulated TFs in the fly. TFs are classified depending on their upregulation in all the species, in two or uniquely in fly. (B) Table showing the number of TFs over the total number of genes in each of the classes described in panel A. (C) Table showing the number of TFs containing an enriched motif in fly DRREs over all the TFs in each of the classes described in panel A.
