# Supplementary material for: Damage-responsive elements in Drosophila regeneration
Source: Genome Res. 2018 Dec;28(12):1852–66. doi: 10.1101/gr.233098.117 (PMC6280756; doi:10.1101/gr.233098.117)
Supplement: Supplemental Material [file supp_gr.233098.117_Supplemental_Table_S6.pdf]

A

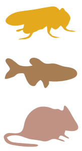	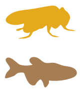	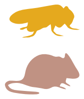	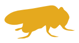	
lilli	**drm**	aop	**cbt**	**phol**
CG13775	vri	CG17612	fu2	**Max**
pdm2	E2f2	CG7099	YL-1	**Deaf1**
elB	Optix	Clamp	abo	**CG11456**
**Dif**	sug	d4	esc	Hr78
Jra	CG6701	Br140	CG6686	CG9727
cyc	twi	**vis**	**crp**	MBD-like
Su(z)12	**pita**	Psc	az2	Irbp
Rbf2	CG7839	mip120	CG1603	**CG31365**
**sr**	**caup**	row	**CG1602**	Dr
**Stat92E**	Eip75B	Dek	**dpn**	ac
**E2f1**	kin17	**CG9890**	**CG1663**	sc
**zfh1**	ato	CG15011	Sox15	crm
Sox100B	bon	**fd68A**	Blos1	Mnt
Med	C15	ash1	**CG8089**	Tip60
Rbf	dys	Gnf1	**CG10543**	CG3815
bi	CG2120	**MTA1-like**	Hmg-2	Nf-YC
**CHES-1-like**	**Her**	**Kdm2**	**CG10321**	CG6769
**sd**		**svp**	NC2alpha	**CG2116**
**Myb**		MBD-R2	**ken**	pad
**disco-r**		**pnr**	**CG11414**	CG3407
		woc	CG2790	
		Ets98B	hng3	
		**wdn**	ecd	
		yem	ERR	
		**CG12054**	Gug	
21	18	26	47	

B

	Shared all	Fly Zebrafish	Fly Mouse	Fly
All genes	268	300	379	1168
TFs	21	18	26	47
%TFs	7,84	6	6,86	4,02

C

	Shared all	Fly Zebrafish	Fly Mouse	Fly
TFs	21	18	26	47
TFs with an enriched motif	9	4	9	16
% TFs with an enriched motif	42,86	22,22	34,61	34,04
